# Comprehensive analyses of mitophagy-related genes and mitophagy-related lncRNAs for patients with ovarian cancer

**DOI:** 10.1186/s12905-023-02864-5

**Published:** 2024-01-13

**Authors:** Jianfeng Zheng, Shan Jiang, Xuefen Lin, Huihui Wang, Li Liu, Xintong Cai, Yang Sun

**Affiliations:** 1 Department of Gynecology, Clinical Oncology School of Fujian Medical University, Fujian Cancer Hospital, Fuzhou, 350014 China; 2 Department of Anesthesiology, The Central hospital of Wenzhou City, 32 Dajian Lane, Wenzhou, 325000 China

**Keywords:** Ovarian cancer, Mitophagy, lncRNA, Prognosis, Immunity

## Abstract

**Background:**

Both mitophagy and long non-coding RNAs (lncRNAs) play crucial roles in ovarian cancer (OC). We sought to explore the characteristics of mitophagy-related gene (MRG) and mitophagy-related lncRNAs (MRL) to facilitate treatment and prognosis of OC.

**Methods:**

The processed data were extracted from public databases (TCGA, GTEx, GEO and GeneCards). The highly synergistic lncRNA modules and MRLs were identified using weighted gene co-expression network analysis. Using LASSO Cox regression analysis, the MRL-model was first established based on TCGA and then validated with four external GEO datasets. The independent prognostic value of the MRL-model was evaluated by Multivariate Cox regression analysis. Characteristics of functional pathways, somatic mutations, immunity features, and anti-tumor therapy related to the MRL-model were evaluated using abundant algorithms, such as GSEA, ssGSEA, GSVA, maftools, CIBERSORT, xCELL, MCPcounter, ESTIMATE, TIDE, pRRophetic and so on.

**Results:**

We found 52 differentially expressed MRGs and 22 prognostic MRGs in OC. Enrichment analysis revealed that MRGs were involved in mitophagy. Nine prognostic MRLs were identified and eight optimal MRLs combinations were screened to establish the MRL-model. The MRL-model stratified patients into high- and low-risk groups and remained a prognostic factor (*P* < 0.05) with independent value (*P* < 0.05) in TCGA and GEO. We observed that OC patients in the high-risk group also had the unfavorable survival in consideration of clinicopathological parameters. The Nomogram was plotted to make the prediction results more intuitive and readable. The two risk groups were enriched in discrepant functional pathways (such as Wnt signaling pathway) and immunity features. Besides, patients in the low-risk group may be more sensitive to immunotherapy (*P* = 0.01). Several chemotherapeutic drugs (Paclitaxel, Veliparib, Rucaparib, Axitinib, Linsitinib, Saracatinib, Motesanib, Ponatinib, Imatinib and so on) were found with variant sensitivity between the two risk groups. The established ceRNA network indicated the underlying mechanisms of MRLs.

**Conclusions:**

Our study revealed the roles of MRLs and MRL-model in expression, prognosis, chemotherapy, immunotherapy, and molecular mechanism of OC. Our findings were able to stratify OC patients with high risk, unfavorable prognosis and variant treatment sensitivity, thus improving clinical outcomes for OC patients.

**Supplementary Information:**

The online version contains supplementary material available at 10.1186/s12905-023-02864-5.

## Background

Ovarian cancer (OC) is the most lethal cancer of the female reproductive system, which is due to the lack of effective screening at the early stage and resistance to chemotherapy as the tumor progresses [1, 2]. The preferred treatment for OC is surgery assisted by the combination of paclitaxel and platinum which prolongates the survival of OC patients [2]. Nevertheless, the survival rate of OC patients with advanced stage is still low, posing a serious threat to women’s lives [1]. Therefore, predicting individual prognosis for OC is important for both patients and gynecologic oncologists.

Cells can remove incomplete or damaged mitochondria through the mechanism of autophagy selectively and the process is called mitophagy [3]. The body can maintain the integrity of mitochondrial function through mitophagy, so as to achieve the purpose of delaying aging and treating diseases [3, 4]. In recent years, mitophagy is found to contribute to OC progression [5, 6]. The specific regulatory mechanism of mitophagy in OC progression may be involved in tumor-associated macrophages [7] and cell stemness [8]. Mitophagy is also involved in anticancer activity of drugs in OC, such as platinum [4, 9–14], EGFR tyrosine kinase inhibitors [15], Janus kinases 1/2 inhibitor [16], pardaxin [17], nanomedicine [5], and epoxycytochalasin H [18]. Despite studies in investigating the role and mechanism of mitophagy in OC, the precise effect of mitophagy in clinical applications remain challenging due to the lack of targetable biomarkers combination.

Long non-coding RNA (lncRNA) refers to a loose RNA transcript with more than 200 nucleotides, which has no protein coding potential [19], and the number of lncRNAs significantly exceeds that of protein-coding genes [19]. Although the functions of lncRNAs in tumorigenesis have been confirmed [19] and our earlier study demonstrated that lncRNA can regulate autophagy in OC [20], little is known about their regulation in mitochondrial function and the mechanism by which lncRNAs regulate mitophagy even remains blank.

Because of the small size and hidden location in the female pelvic cavity, early diagnosis of OC is extremely challenging [1]. Currently, the most commonly used tumor marker for OC screening in clinical practice is Carbohydrate Antigen 125 (CA125) [21] and Human Epididymis Protein 4 (HE4) [22]. Given that other benign diseases can also cause elevated serum biomarkers, the diagnostic specificity and sensitivity of using serum CA125 or HE4 alone are not high [23]. Existing studies have attempted to establish prognostic models for patients with OC based on clinicopathologic characteristics. For instance, the Risk of Ovarian Malignancy Algorithm (ROMA) model incorporated both serum CA125 and HE4, nevertheless, the model did not fully address the challenge of detecting OC with high risk [23].

More and more studies show that gene expression profiles can be used to identify many important prognostic genes in various types of cancer and to map prognostic related molecular models [24, 25]. Based on high-throughput technologies and data sharing, cancer research has entered the era of big data due to large-scale multi-omics data accumulated in The Cancer Genome Atlas (TCGA) [26] and Gene Expression Omnibus (GEO) databases [27]. Bioinformatics is an emerging interdisciplinary subject used for analyzing biological information [28], which takes computer as a tool (mainly R packages) [29]. The application of big data from TCGA and GEO databases based on bioinformatics allows us to evaluate the predictive value of mitophagy-related lncRNA (MRL) combinations for OC patients.

The packages in R language software can be used for data mining and statistical analysis [30]. Herein, we mainly utilized R packages to carry out comprehensive analyses of mitophagy-related genes (MRGs) and MRLs for patients with OC. Using weighted co-expression network analysis (WGCNA) and least absolute shrinkage and selection operator (LASSO) Cox regression analysis, we analyzed the landscape of MRGs and MRLs comprehensively. The reliable MRL-model to predict overall survival (OS) and therapeutic strategies was constructed. Our data showed that the MRL-model was associated with immunity characteristics, tumor mutational burden (TMB), immunotherapy, and chemotherapeutic drug sensitivity.

## Methods

### Data collection

The processed data were extracted from UCSC-Xena (https://xenabrowser.net/datapages/) [31]. The Ensemble Gene was converted into Gene Symbol based on gene annotation information in GENCODE [32]. The low-expression mRNAs and lncRNAs were filtered. Collectively, 417 OC samples with expression profiles and prognostic information from TCGA were included. Besides, 88 normal ovarian tissues from GTEx were obtained for identification of differentially expressed genes. We also retrieved four OC datasets that had lncRNA expression profiles and prognostic information from GEO database (https://www.ncbi.nlm.nih.gov/geo/) [27], including 268 OC cases. We selected the dataset from the GPL570 Affymetrix Human Genome U133 Plus 2.0 Array to annotate as many lncRNAs as possible. MRGs were screened from GeneCards (https://www.genecards.org) [33] based on their relevance score. Furthermore, the somatic mutations were generated with Mutation Annotation Format (MAF) using the “maftools” package (Version 2.16.0) [34].

### Differentially expressed genes screening

Linear regression and Empirical Bayesian [35] were able to shrink the analyzed variances toward a common estimate and the method was conducted using “limma” package (Version 3.10.3) [36] to screen out the differentially expressed MRGs and lncRNAs. Benjamini-Hochberg was used for multiple test correction to obtain greater power relative based on False Discovery Rate (FDR) [37]. The threshold of screening differentially expressed genes was set as adjusted *P* < 0.05 and |logFC| > 0.5.

### Prognostic genes screening

The “survminer” package (Version 0.4.3) was used to determine the optimal cut-point based on the expression of genes, survival time and survival state. The prognostic genes were screened out based on Kaplan-Meier (K-M) curves and logRank test.

### MRLs screening based on WGCNA

We used the “WGCNA” package [38] (Version 1.61) to analyze the expression matrix of lncRNAs, so as to identify highly synergistic lncRNA modules. Firstly, a series of power was set to calculate the square value of correlation coefficient between connectivity k and p(k) and the average connectivity under each power value. The power value whose square value of correlation coefficient reached above 0.85 for the first time was selected. Secondly, based on dynamic pruning and clustering methods, we aggregated highly correlated lncRNAs into modules (correlation coefficient > 0.8). Finally, the correlation between modules and the prognostic MRGs was calculated, and the lncRNA modules associated with multiple MRGs were identified. We defined the modules with the most obvious positive or negative correlation with multiple MRGs as the key modules, and the lncRNAs in these modules were MRLs.

### Establishment of the MRL-model

After obtaining prognostic MRLs, we applied the high-dimensional index regression method of “glmnet” R package (Version 2.0–18), LASSO Cox regression analysis, to screen the combination of prognostic MRLs by utilizing a penalty proportional to the contraction of the regression coefficient based on 20-fold cross-validation analysis, thus addressing multicollinearity [39]. The regression coefficient and the expression level of each MRL was applied to calculate the risk score and construct the MRL-model as follows:

$$Risk\ Score=\sum {\beta}_{lncRNA}\times {Exp}_{lncRNA}$$

Herein, β_lncRNA_ was the LASSO regression coefficient of the MRL, and Exp_lncRNA_ represented the expression value of MRL. The highly correlated MRLs were excluded to prevent the MRL-model from overfitting.

### Validation of the MRL-model

We included four external datasets that had lncRNA expression profile and prognostic information to validate the model: GSE19829 (28 OC samples), GSE26193 (107 OC samples), GSE30161 (58 OC samples), and GSE63885 (75 OC samples). The batch effects of the four external datasets were removed by “sva” R package (Version 3.48.0) [40]. The β_lncRNA_ was first generated based on TCGA training dataset and the risk score of the GEO validation datasets was calculated based on the formula described above. TCGA training and GEO validation datasets were divided into high-risk group (risk score higher than threshold value), or low-risk group (risk score lower than threshold value) based on the threshold value (median of risk score). K-M curves were used to evaluate the survival outcomes of risk groups for TCGA training and GEO validation datasets, thus validating the effectiveness of predicting prognosis.

### Establishment of the nomogram based on MRL-model

We conducted Univariate Cox regression analysis to assess the prognostic value of MRL-model and clinicopathological parameters. Multivariate Cox regression analysis was further implemented to evaluate and validate their independent prognostic value in TCGA training and GEO validation datasets. Subsequently, the “rms” package (Version 6.7.0) was applied to establish the Nomogram based on MRL-model and clinicopathological parameters [41]. The Nomogram was validated by discrimination and calibration with B = 1000 resampling optimism added to describe the relationship between the actual and the predicted OS probability of the Nomogram, thus evaluating the consistency of the MRL-model. The closer the predicted curve is to 45°, the better the prediction ability.

### Quantitative real-time PCR

A total of 30 OC and 10 normal tissues were collected after approving by Ethics Committee of Clinical Oncology School of Fujian Medical University, Fujian Cancer Hospital. The samples obtained were pathologically confirmed as OC or ovarian tissues. Quantitative Real-time PCR analysis (SuperReal PreMix Plus from Tiangen Biotech, Beijing, China) was carried out after extracting total RNA (TRNzol Universal Reagent from Tiangen Biotech, Beijing, China) and reverse transcription (FastKing gDNA Dispelling RT SuperMix from Tiangen Biotech, Beijing, China). The sequence of lncRNA was obtained from LNCipedia (https://lncipedia.org/) [42]. The primers of the lncRNAs were designed and provided by Sangon Biotech (Shanghai, China).

### Analysis of functional pathways

The protein-protein interaction (PPI) network was established using STRING (https://string-db.org/) [43] and Cytoscape (Version 3.4.0) [44]. Gene set enrichment analysis (GSEA) was performed in high-risk group versus low-risk group using “GSEA” (Version 4.3.2) [45]. The background gene set was the pathway set in MsigDB molecular label database [46].

### Analysis of immunity features

The carcinogenesis of OC is strongly correlated with the immune microenvironment [47]. Utilizing single-sample gene set enrichment analysis (ssGSEA), we calculated enrichment fraction of 28 immune cells using gene set variation analysis (GSVA, Version 1.48.3) to indicate the relative abundance of each tumor microenvironment-infiltrated cell [48]. In addition, three algorithms, CIBERSORT (Cell-type Identification By Estimating Relative Subsets Of RNA Transcripts, Version 0.1.0) [47], xCELL (Version 1.1.0) [49], MCPcounter (Microenvironment Cell Populations-counter, Version 1.2.0) [50], were used to characterize the cellular composition of complex tissues according to corresponding literature. Further, we estimated immune and stromal scores using ESTIMATE (Estimation of STromal and Immune cells in MAlignant Tumor tissues using Expression data) algorithm (Version 1.1.7) to indicate the presence of stromal and immune cells [51].

### Analysis of therapy

We predicted potential responses to immune checkpoint blockade (ICB) using the Tumor Immune Dysfunction and Exclusion (TIDE) tool (http://tide.dfci.harvard.edu/) [52]. Through contrasting gene expression profiles of OC and dataset of immunotherapy, we compared the discrepancy between the two risk groups in immunotherapy using submap and the *P* value was Bonferroni corrected [53]. The reactivity of chemotherapy drugs were extracted from the Genomics of Drug Sensitivity in Cancer (GDSC) database (https://www.cancerrxgene.org/) [54] and we used “pRRophetic” package (Version 0.5) [55] to analyze cell line expression profiles and OC gene expression profiles by constructing ridge regression model to assess IC50 levels of drugs.

### Construction of ceRNA network

Pearson correlation coefficient (correlation coefficient > 0.2) between mRNAs and lncRNAs was calculated and FDR value (FDR < 0.05) was obtained from Benjamini-Hochberg correction. The local software miranda (Version 3.3a) [56] was used to screen the lncRNA-mRNA pairs (Score ≥ 140 and Energy≤ − 20). We used miRWalk3.0 (http://mirwalk.umm.uni-heidelberg.de/search_genes/) [57] to obtain the miRNA-mRNA pairs which had been verified by experiment. Further, lncRNAs and mRNAs regulated by the same miRNA with positive co-expression relationship were screened to establish the ceRNA (competing endogenous RNA) network. We used Cytoscape software (Version 3.4.0) for network graph construction [44]. The Degree Centrality of network node were analysed using CytoNCA plug-in (Version 2.1.6) [58].

### Statistical analysis

The statistical analysis and graph visualization were performed by using R programming language [59, 60] or GraphPad Prism. The software, packages and their versions used for statistical analysis were listed in Supplementary Table S1. The genes with prognostic value were identified based on the hazard ratio (HR) and 95% confidence interval (CI). K-M curves and log-rank test were applied to contrast the survival outcome between two subgroups. Univariate and Multivariate Cox analyses were conducted to determine the independent prognostic value. Wilcox test was used to compare the immune characteristic or drug sensitivity between two groups. The two-tailed *P* lower than 0.05 was considered statistically significant.

## Results

**Fig. 1:**
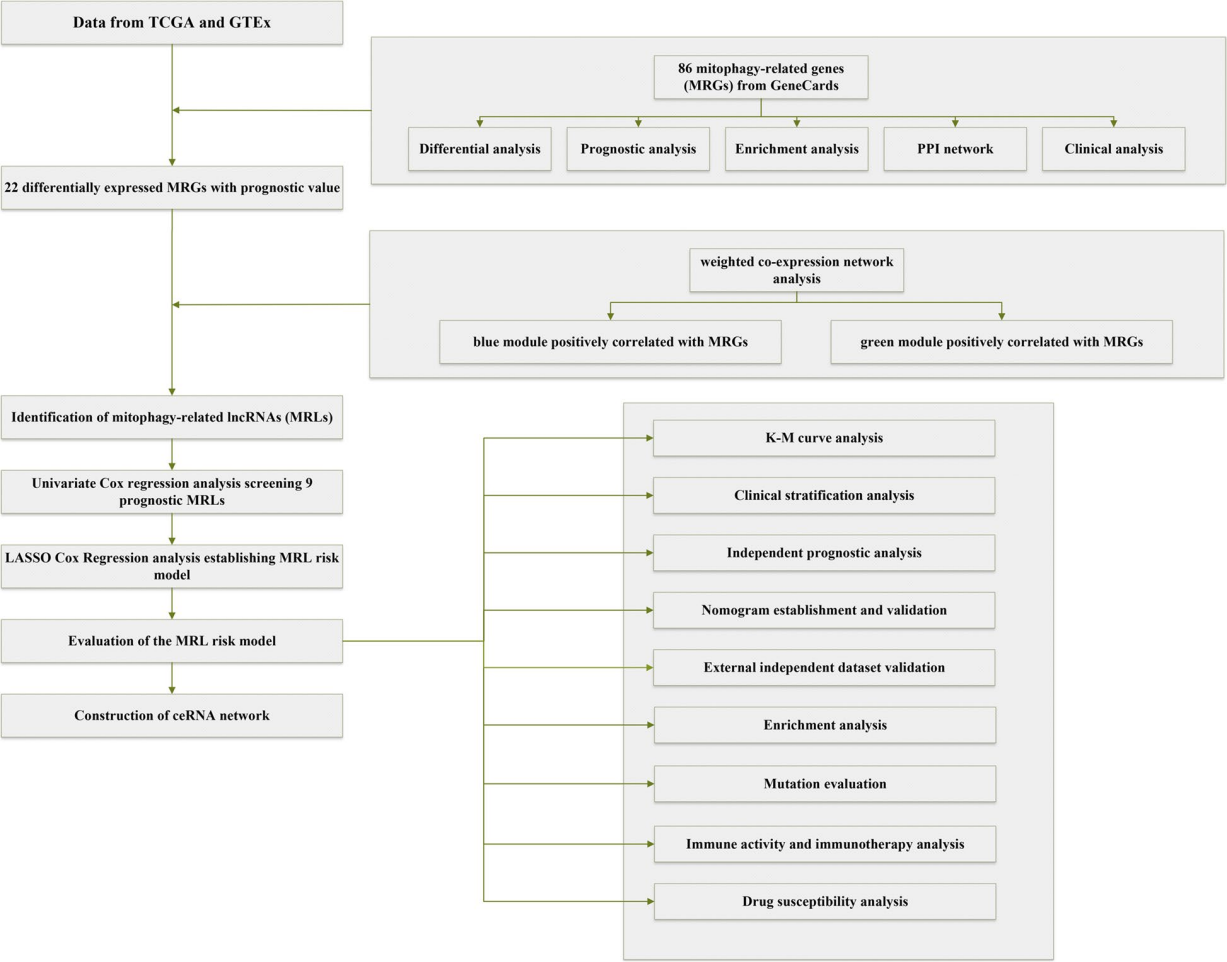
The flowchart outlining the main design of our study

The research flowchart was plotted to summarize the main design of our study (Fig. 1).

### Differentially expressed and prognostic genes screening

**Fig. 2:**
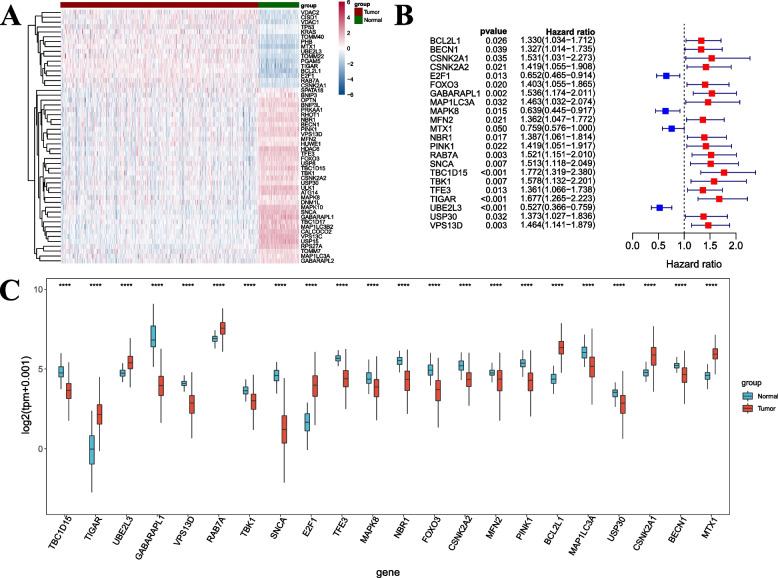
Differentially expressed and prognostic MRGs screening. **A** Heatmap showing the gene expression of the 52 differentially expressed MRGs between OC and normal tissues. **B** The forest map showing the results of prognosis analysis of 22 differentially expressed MRGs with prognostic value. The values in parentheses represent 95% confidence intervals of hazard ratio. **C** Box diagram showing the 22 prognostic MRGs expression between normal and tumor groups. ∗∗∗∗*P* < 0.0001

Compared with normal, there were 52 MRGs differentially expressed in OC (adjusted *P* < 0.05 and |logFC| > 0.5) (Fig. 2A). Through prognostic analysis, we found that 22 of the 52 MRGs were significantly correlated with the prognosis (Fig. 2B). Among the 22 prognostic MRGs, there were four MRGs correlated with favorable prognosis (HR < 1), including E2F1, MAPK8, MTX1, and UBE2L3. In contrast, the remaining 18 MRGs were associated with a poor prognosis (HR > 1), including BCL2L1, BECN1, CSNK2A1, CSNK2A2, FOXO3, GABARAPL1, MAP1LC3A, MFN2, NBR1, PINK1, RAB7A, SNCA, TBC1D15, TBK1, TFE3, TIGAR, USP30, and VPS13D. The box diagram visually demonstrated the expression differences of these 22 prognostic MRGs between OC and normal tissues (Fig. 2C).

To further observe the relationship between the 22 prognostic MRGs and clinicopathological parameters, box plots for each MRG were drawn between different clinical groups. We found that TBC1D15 (*P* < 0.05), UBE2L3 (*P* < 0.05), VPS13D (*P* < 0.05), TFE3 (*P* < 0.01), NBR1 (*P* < 0.01), MFN2 (*P* < 0.01), PINK1 (*P* < 0.05), USP30 (*P* < 0.05), and CSNK2A1 (*P* < 0.01) was associated with the stage of OC (Supplementary Fig. S1A). Most of the MRGs were not significantly different among other clinical factors, except SNCA (*P* < 0.05) and E2F1 (*P* < 0.05) in Grade (Supplementary Fig. S1B), CSNK2A2 (*P* = 0.02) in Age (Supplementary Fig. S1C), TIGAR (*P* < 0.05) and MTX1 (*P* < 0.05) in Macroscopic disease (Supplementary Fig. S1D).

In summary, the differentially expressed and prognostic MRGs can be used as diagnostic markers to identify cancer and non-cancer as well as different clinical stages. These MRGs are expected to be involved in OC progression and deserve further study.

### The interactions among MRGs

**Fig. 3:**
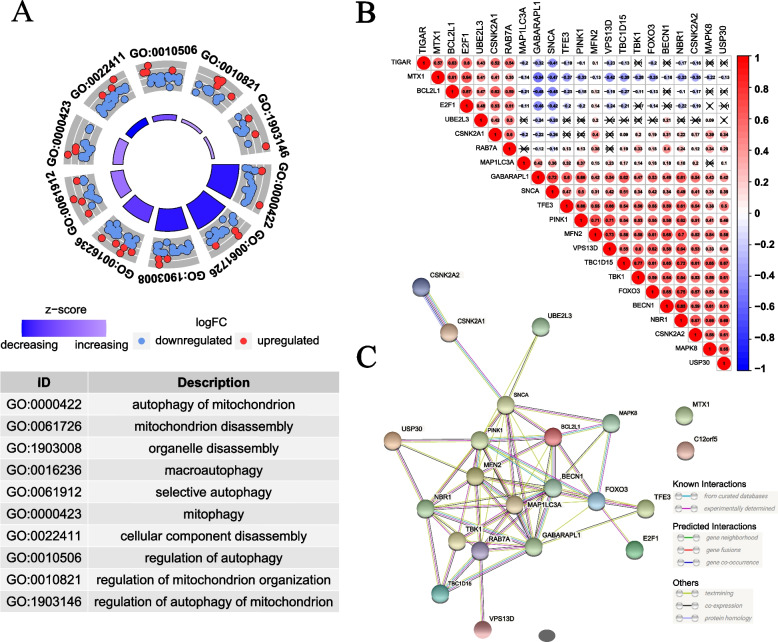
The interactions among MRGs. **A** GO enrichment analysis of MRGs showing their significant function in mitophagy. **B** The correlations among the expression of the 22 prognostic MRGs. The color and number of the circle represent the correlation coefficient. The crossed symbol indicates no statistical significance. **C** PPI network encoded by 22 prognostic MRGs showing their interactions

Gene Ontology (GO) enrichment analysis revealed that the MRGs were enriched in mitophagy, mitochondrion disassembly, organelle disassembly, macroautophagy, cellular component disassembly, regulation of mitochondrion organization, and so on (Fig. 3A), suggesting that these MRGs were indeed involved in mitophagy and their biological implications for wet experiments. Interestingly, the correlations among the expression of the 22 prognostic MRGs were mostly positive, and CSNK2A2 and NBR1 (*P* < 0.05, Cor = 0.85) were the most positively correlated gene pair (Fig. 3B), which further hinted at their similarity in biological functions. To further explore the interactions of these 22 MRGs, the PPI analysis was performed (Fig. 3C). By ranking the degree in the PPI network, we could find that BECN1, GABARAPL1, PINK1, SNCA, MAP1LC3A, MEN2, and NBR1, FOXO3, RAB7A, and BCL2L1 were the top nine hub genes (Supplementary Fig. S2), indicating that these MRGs play a more prominent role in mitophagy in OC. Genetic mutations of the majority of MRGs were not detected in OC samples, except TP53, HUWE1, and VPS13C (Supplementary Fig. 3), hence most MRGs are wild-type in OC.

### MRLs screening based on WGCNA

**Fig. 4:**
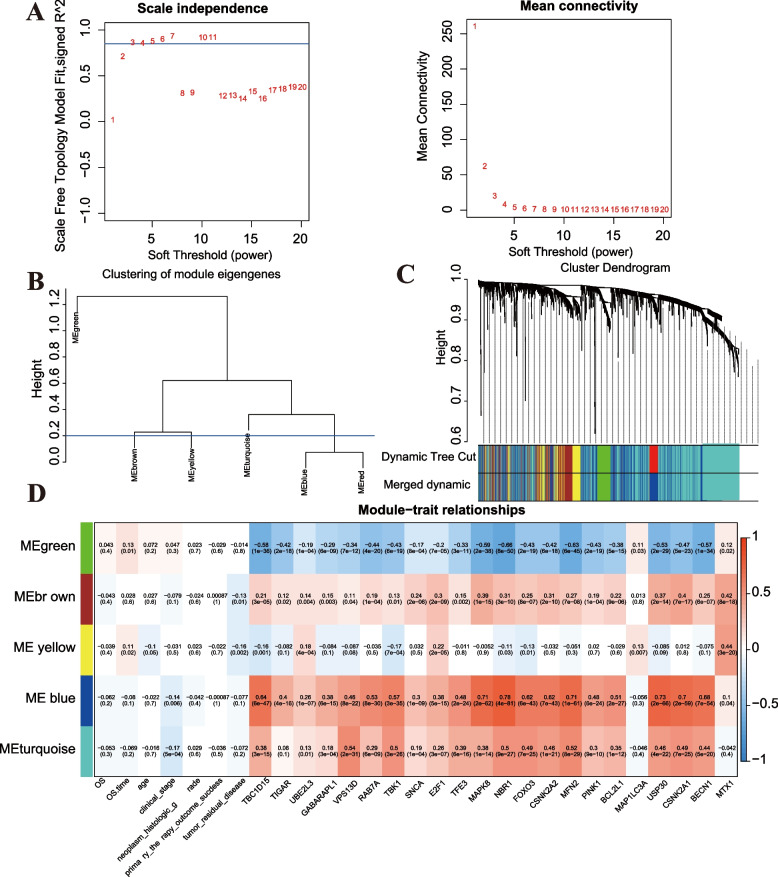
MRLs screening based on WGCNA. (**A**) WGCNA power result. Left: Soft Threshold (power) represents the weight, and the ordinate represents the square value of the correlation coefficient between connection degree k and p(k). Right: Soft Threshold (power) represents the weight, and the ordinate represents the average connection. It is generally required that the power when the square value of the correlation coefficient between k and p(k) reaches 0.8 for the first time is taken as the β value, which can be seen as β = 3. (**B**) The module clustering result diagram. The vertical axis represents the difference coefficient, and the blue line represents the difference coefficient of 0.2. (**C**) Systematic cluster tree of genes and gene modules generated by dynamic clipping method. Different colors represent different genetic modules. (**D**) Heatmap of the correlation between module eigengenes and 22 prognostic MRGs of OC. Each cell contains the correlation coefficient and *P* value. The top number represents the correlation coefficient, and the parenthesis number represents the significance *P* value

We conducted WGCNA analysis on the lncRNAs obtained. Firstly, the soft threshold was set to 9 (Fig. 4A). We set β = 3 since the power when the square value of the correlation coefficient between k and p(k) reaches 0.8 for the first time. Based on dynamic pruning and clustering, high correlation genes were aggregated into modules. Then we clustered these modules and merge modules with a correlation coefficient greater than 0.8. To wit, modules with a coefficient of dissimilarity less than 0.2 (Fig. 4B), and finally integrated into five modules (Fig. 4C). The correlation between 22 prognostic MRGs and module eigengene were further calculated. The blue module (containing 369 lncRNAs) revealed the strongest positive correlation with most MRGs, while the green module (containing 70 lncRNAs) showed the strongest negative correlation with most MRGs (Fig. 4D). Therefore, the subsequent analysis was mainly according to the lncRNAs in the two modules. We defined these lncRNAs as MRLs. After the above complex comprehensive analysis, we reliably obtained MRLs closely related to mitophagy, which laid the foundation for the following studies.

### Screening of prognostic MRLs

**Fig. 5:**
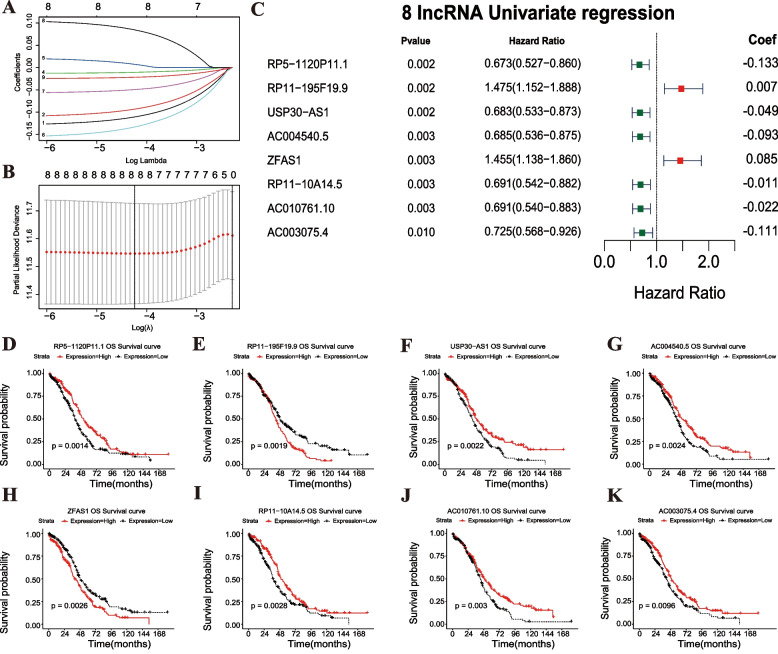
Screening of prognostic MRLs. **A** LASSO regression of the eight optimal MRLs. **B** Cross-validation for tuning the parameter selection and showing confidence interval under each lambda the in the LASSO regression. The two dashed lines indicate two special λ values: λ_min_ on the left and λ_1se_ on the right. The λ values between these two values were considered to be appropriate. The model constructed by λ_1se_ was the simplest, that was, it used a small number of genes, while λ_min_ had a higher accuracy rate and used a larger number of genes. Hence, λ_min_ was selected to build the model for accuracy in our study. **C** The forest map showing the results of Cox regression analysis and LASSO regression coefficient of the eight optimal MRLs. The values in parentheses represented 95% confidence intervals. **D**-**K** Survival analysis of the eight optimal MRLs for RP5-1120P11.1 (**D**), RP11-195F19.9 (**E**), USP30-AS1 (**F**), AC004540.5 (**G**), ZFAS1 (**H**), RP11-10A14.5 (**I**), AC010761.10 (**J**), and AC003075.4 (**K**). The *P* values are tested by log-rank

According to the MRLs in the blue and green modules mentioned above, we performed Univariate Cox regression analysis first. Our data showed that nine MRLs were significantly associated with survival prognosis. Then, eight optimal lncRNA combinations were screened by LASSO Cox Regression algorithm combining the expression value of MRLs, survival time and survival state (Fig. 5A-B). The forest map revealed the results of LASSO regression coefficient and Cox regression analysis of the eight optimal MRLs (Fig. 5C), including RP5-1120P11.1 (*P* = 0.002; HR = 0.673, 95% CI:0.527–0.860; Coef = − 0.133), RP11-195F19.9 (*P* = 0.002; HR = 1.475, 95% CI:1.152–1.888; Coef = 0.007), USP30-AS1 (*P* = 0.002; HR = 0.683, 95% CI:0.533–0.873; Coef = − 0.049), AC004540.5 (*P* = 0.003; HR = 0.685, 95% CI:0.536–0.875; Coef = − 0.093), ZFAS1 (*P* = 0.003; HR = 1.455, 95% CI:1.138–1.860; Coef = − 0.085), RP11-10A14.5 (*P* = 0.003; HR = 0.691, 95% CI:0.542–0.882; Coef = − 0.011), AC010761.10 (*P* = 0.003; HR = 0.691, 95% CI:0.540–0.883; Coef = − 0.022), and AC003075.4 (*P* = 0.010; HR = 0.725, 95% CI:0.568–0.926; Coef = − 0.111). K-M curves were drawn to evaluate the association between the expression levels of the eight optimal MRLs and OC survival prognosis, including RP5-1120P11.1 (log-rank test *P* = 0.0014), RP11-195F19.9 (log-rank test *P* = 0.0019), USP30-AS1 (log-rank test *P* = 0.0022), AC004540.5 (log-rank test *P* = 0.0024), ZFAS1 (log-rank test *P* = 0.0026), RP11-10A14.5 (log-rank test *P* = 0.0028), AC010761.10 (log-rank test *P* = 0.003), and AC003075.4 (log-rank test *P* = 0.0096) (Fig. 5D-K). In summary, except for RP11-195F19.9 and ZFAS1, which were associated with poor prognosis, the remaining MRLs were associated with better prognosis of OC. Therefore, we have screened out the optimal combination of MRLs that are involved in OC progression and will build a prognostic MRL-model to calculate risk score for OC based on the results.

### Identification and validation of the MRL-model

**Fig. 6:**
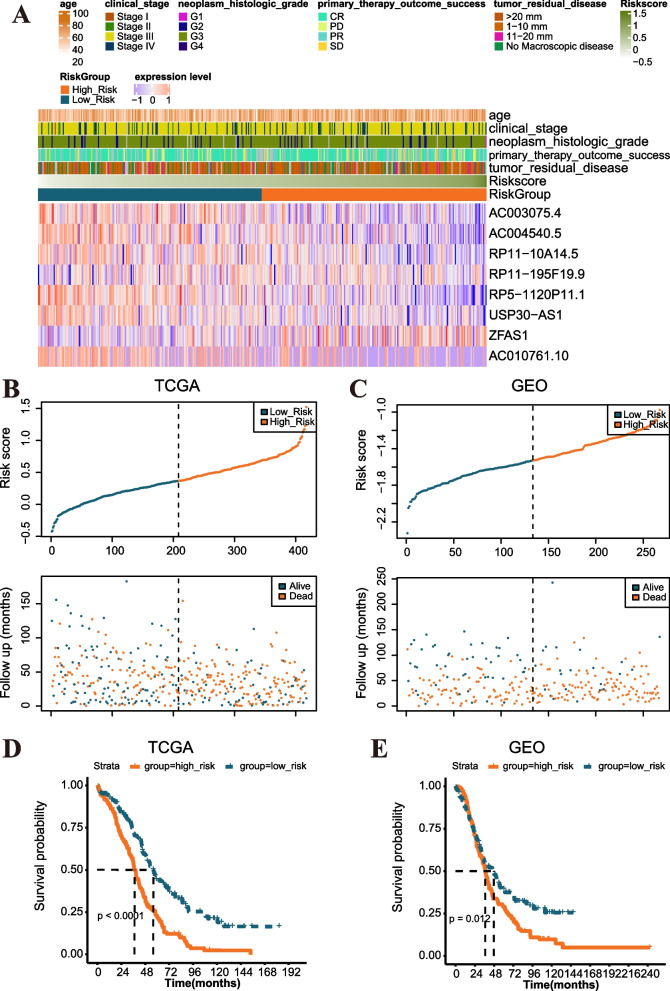
Identification and validation of the MRL-model. **A** Heatmap of the associations among the expression levels of the eight MRLs, risk score and clinicopathological parameters. **B, C** Distribution of risk scores and survival status of OC patients in TCGA training (**B**) and GEO validation (**C**) datasets. **D, E** Kaplan-Meier survival curves show survival probability of high-risk or low-risk in TCGA training (**D**) and GEO validation (**E**) datasets. The *P* values are tested by log-rank

The expression levels of the eight optimal MRLs varied in different samples with risk score and clinical information as shown in Fig. 6A. We could see that high expression of ZFAS1 and RP11-195F19.9 was associated with high risk score, but the opposite was true for the remaining six MRLs. Using the same regression coefficient, the risk score of TCGA training and GEO validation datasets was calculated based on the formula described in Methods section. Patients with risk score higher than the median were included in high-risk group, otherwise, they were included in low-risk group. Figure 6B, C illustrated the distribution of risk score in the two risk groups, and the good prognosis of patients in the low-risk group was observed in both the TCGA training (log-rank test *P* < 0.0001) (Fig. 6D) and GEO validation (log-rank test *P* = 0.012) (Fig. 6E) datasets, thus proving the accuracy of the validation.

**Fig. 7:**
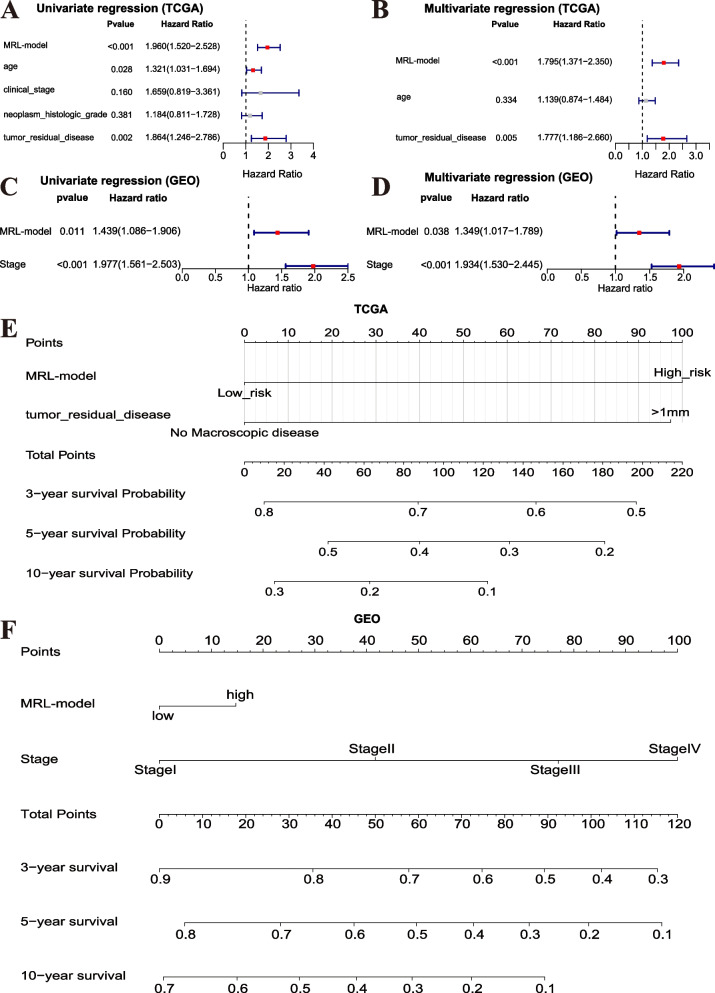
Establishment of the Nomogram based on MRL-model. **A** The Univariate Cox regression analysis for TCGA training dataset. **B** The Multivariate Cox regression analysis for TCGA training dataset. **C** The Univariate Cox regression analysis for GEO validation dataset. **D** The Multivariate Cox regression analysis for GEO validation dataset. The values in parentheses represent 95% confidence intervals. **E** The Nomogram model based on MRL-model and clinicopathological parameters for TCGA training dataset. **F** The Nomogram model based on MRL-model and clinicopathological parameters for GEO validation dataset

Based on Univariate Cox regression analysis, the MRL-model was proved to be a prognostic marker (*P* < 0.001; HR = 1.960, 95% CI:1.520–2.528) in TCGA training dataset (Fig. 7A). Besides, even though we performed Multivariate Cox regression analysis combining the MRL-model and clinicopathological parameters, the MRL-model remained a significant independent predictive predictor (*P* < 0.001; HR = 1.795, 95% CI:1.371–2.350) in TCGA training dataset (Fig. 7B). We further carried out Univariate (Fig. 7C) and Multivariate (Fig. 7D) Cox regression analyses for GEO validation datasets to validate the above results. Our data revealed that the MRL-model was also a prognostic marker (*P* = 0.011; HR = 1.439, 95% CI:1.086–1.906) and a significant independent predictive predictor (*P* = 0.038; HR = 1.349, 95% CI:1.017–1.789) in the validation datasets. The Nomogram was plotted to make the prediction results more intuitive and readable for TCGA training (Fig. 7E) and GEO validation (Fig. 7F) datasets. The Nomogram was further validated by discrimination and calibration to describe the relationship between the actual OS probability and the predicted OS probability. We observed that the predicted curve was adjacent to 45° in TCGA training (Supplementary Fig. S4A) and GEO validation (Supplementary Fig. S4B) datasets, indicating the favorable prediction ability.

Ulteriorly, we implemented stratification analyses based on clinicopathological parameters to further validate the effectiveness of the MRL-model in predicting OC prognosis. We observed that OC patients in the high-risk group still had the unfavorable survival in consideration of Age < 60 (log-rank test *P* < 0.001) (Supplementary Fig. S5A), Age > =60 (log-rank test *P* < 0.001) (Supplementary Fig. S5B), Stage I-II (log-rank test *P* = 0.581) (Supplementary Fig. S5C), Stage III-IV (log-rank test *P* < 0.001) (Supplementary Fig. S5D), Grade I-II (log-rank test *P* = 0.348) (Supplementary Fig. S5E), Grade III-IV (log-rank test *P* < 0.001) (Supplementary Fig. S5F), Tumor Residual Disease 1-10 mm (log-rank test *P* = 0.002) (Supplementary Fig. S5G), Tumor Residual Disease > 10 mm (log-rank test *P* = 0.053) (Supplementary Fig. S5H), White (log-rank test *P* < 0.001) (Supplementary Fig. S5I) and Nonwhite (log-rank test *P* = 0.113) (Supplementary Fig. S5J).

To sum up, the MRL-model is a reliable prognostic risk stratification tool for OC and is worthy of further large sample validation for clinical implications.

### Quantitative real-time PCR

The eight optimal MRLs in the MRL-model were examined to compare the difference between normal and cancer tissues via Quantitative Real-time PCR experiments. In TCGA dataset (Supplementary Fig. S6A-H), AC003075.4 (*P* < 0.0001), AC004540.5 (*P* < 0.0001), AC010761.10 (*P* < 0.0001), RP5-1120P11.1 (*P* < 0.0001), RP11-10A14.5 (*P* < 0.0001), USP30-AS1 (*P* < 0.0001) were highly expressed in OC, conversely, the expression levels of RP11-195F19.9 (*P* < 0.0001) and ZFAS1 (*P* < 0.0001) were low in OC tissues. This difference in expression was also observed in our cohort (Supplementary Fig. S6I-P): AC003075.4 (*P* = 0.0834), AC004540.5 (*P* < 0.01), AC010761.10 (*P* < 0.05), RP5-1120P11.1 (*P* < 0.05), RP11-10A14.5 (*P* < 0.05), RP11-195F19.9 (*P* < 0.05), USP30-AS1 (*P* < 0.01) and ZFAS1 (*P* < 0.01). Our results further confirm the potential of these MRLs as diagnostic markers for OC. However, more sample size and prognostic follow-up information are needed in future studies.

### Evaluation of functional pathways for the MRL-model

**Fig. 8:**
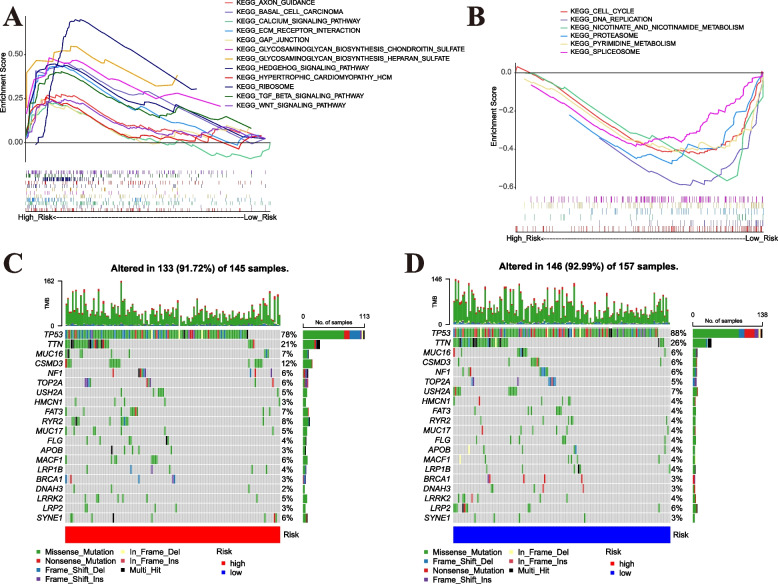
Evaluation of functional pathways and mutation for the MRL-model. **A**, **B** GSEA enrichment analysis of the status of special biological pathways in high-risk group and low-risk group. **C**, **D** The waterfall plot of somatic mutation features established with high- (**C**) and low- (**D**) risk groups. Each column represented an individual patient. The upper barplot showed TMB, the number on the right indicated the mutation frequency in each gene. The right barplot showed the proportion of each variant type

GSEA enrichment analysis was performed on KEGG pathway in high-risk group versus low-risk group based on GSEA software. There were 18 KEGG pathways which were significantly enriched in low-risk group and14 KEGG pathways were significantly enriched in high-risk group. Due to the large number of results, only *P* value< 0.01 was shown in Fig. 8A, B, including 12 enrichment pathways in the high-risk group (*P* < 0.01) (Fig. 8A) and six enrichment pathways in the low-risk group (*P* < 0.01) (Fig. 8B). We could find that the high-risk group was enriched in some classic tumor-related signaling pathways, for example, Wnt signaling pathway, TGF-beta signaling pathway, and Hedgehog signaling pathway (Fig. 8A). The low-risk group was mainly enriched in metabolic pathways, such as nicotinate and nicotinamide metabolism, pyrimidine metabolism (Fig. 8B). These enriched pathways could provide new insights into the underlying biological implications of OC patients with different risk stratifications.

### Evaluation of mutation for the MRL-model

Our study revealed that TMB was higher in low-risk group than in high-risk group (*P* < 0.05), implying that patients with lower risk score may benefit from immunotherapy (Supplementary Fig. S7A). The Spearman correlation coefficient between risk score and TMB was negative (r = − 0.1279, *P* = 0.0294), demonstrating that TMB was negatively associated with risk score (Supplementary Fig. S7B). The distribution variations of the somatic mutations between the two risk groups were also analyzed. The top 20 mutated genes in the two risk groups were TP53 (78, 88%), TTN (21, 26%), MUC16 (7, 6%), CSMD3 (12, 6%), NF1 (6, 6%), TOP2A (6, 5%), USH2A (5, 7%), HMCN1 (3, 4%), FAT3 (7, 4%), RYR2 (8, 4%), MUC17 (5, 4%), FLG (4, 4%), APOB (3, 4%), MACF1 (6, 4%), LRP1B (4, 4%), BRCA1 (3, 3%), DNAH3 (2, 3%), LRRK2 (5, 4%), LRP2 (3, 6%), and SYNE1 (6, 3%) (Fig. 8C-D). OC patients with higher risk score had observably lower frequencies of TP53 and TTN (Fig. 8C). However, the mutated levels of CSMD3 and RYR2 were opposite (Fig. 8D). Overall, patients in the low-risk group had a greater mutation rate, and the lower risk score may be an indicator that immunotherapy is effective.

### Analysis of immunity features and immunotherapy for the MRL-model

**Fig. 9:**
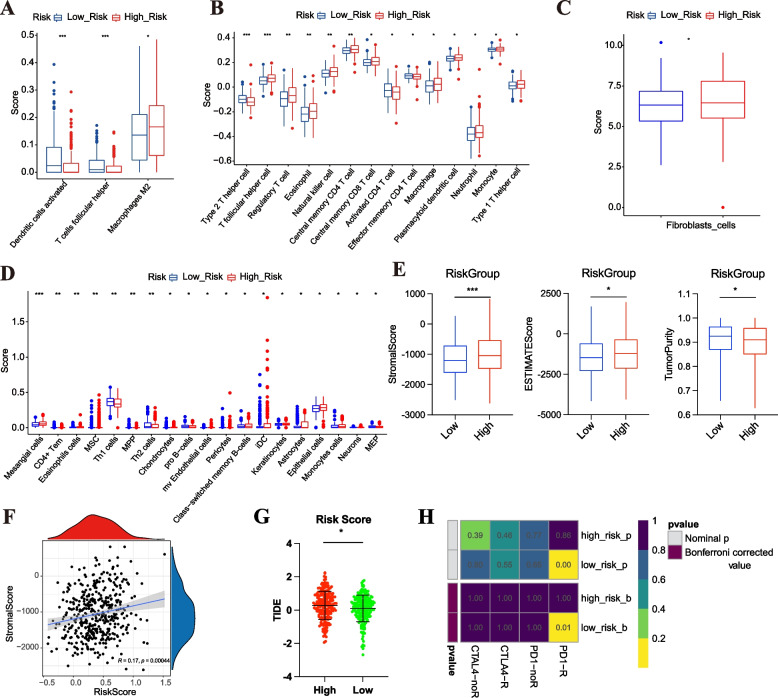
Analysis of immunity features and immunotherapy for the MRL-model. **A**-**E** Analysis of immune activity between the two risk groups using five algorithms: CIBERSORT (**A**), ssGSEA (**B**), MCPcounter (**C**), xCELL (**D**), and ESTIMATE (**E**). **F** Correlations between risk scores and stromal scores. **G** TIDE score of OC patients in the two risk groups. **H** Subclass mapping showing the difference in immunotherapy between the two risk groups

To further explore the relationship between immune features and MRL-model, five algorithms, including CIBERSORT (Fig. 9A), ssGSEA (Fig. 9B), MCPcounter (Fig. 9C), xCELL (Fig. 9D), and ESTIMATE (Fig. 9E), were used to analyze immune features for the MRL-model. The results suggested that OC patients in the two risk groups differed at the level of immune cells (Fig. 9A-D). Higher risk score correlated strongly with higher stromal (*P* < 0.001) and estimated (*P* < 0.05) scores, while lower risk score correlated with higher tumor purity (*P* < 0.05) (Fig. 9E). Risk score was shown to be significantly positively correlated with Stromal Score (R = 0.17, *P* = 0.00044) (Fig. 9F). However, there was no significant correlation between risk score and ESTIMATE Score (R = 0.089, *P* = 0.071), Immune Score (R = -0.004, *P* = 0.93), and Tumor Purity (R = -0.089, *P* = 0.071) (Supplementary Fig. S8A-B). Furthermore, we investigated the association between the risk score and seven immune checkpoints. Four immune checkpoints, including CD274 (*P* < 0.05), CD47 (*P* < 0.001), LAG3 (*P* < 0.01), and VTCN1 (*P* < 0.001) were under-expressed in high-risk group (Supplementary Fig. S9). Nevertheless, the expression values of the remaining immune checkpoints did not differ between the two risk groups. Higher TIDE score was not only associated with worse immune checkpoint inhibition therapy, but also with worse survival with anti-CTLA4 and anti-PD1 therapy. From Fig. 9G, we could find that TIDE score of OC patients in the low-risk group was lower than that in high-risk group, suggesting that OC patients with low risk score were more sensitive to immune checkpoint blockade therapy (*P* < 0.05). In addition, through the results of subclass mapping, we found that OC patients in the low-risk group may be more sensitive to PDL1 response (Bonferroni corrected *P* = 0.01) (Fig. 9H). Therefore, we could conclude that patients in the low-risk group identified by the MRL-model may be more sensitive to immunotherapy, which may provide a reference for clinical immunotherapy of OC.

### Analysis of drug sensitivity for the MRL-model

**Fig. 10:**
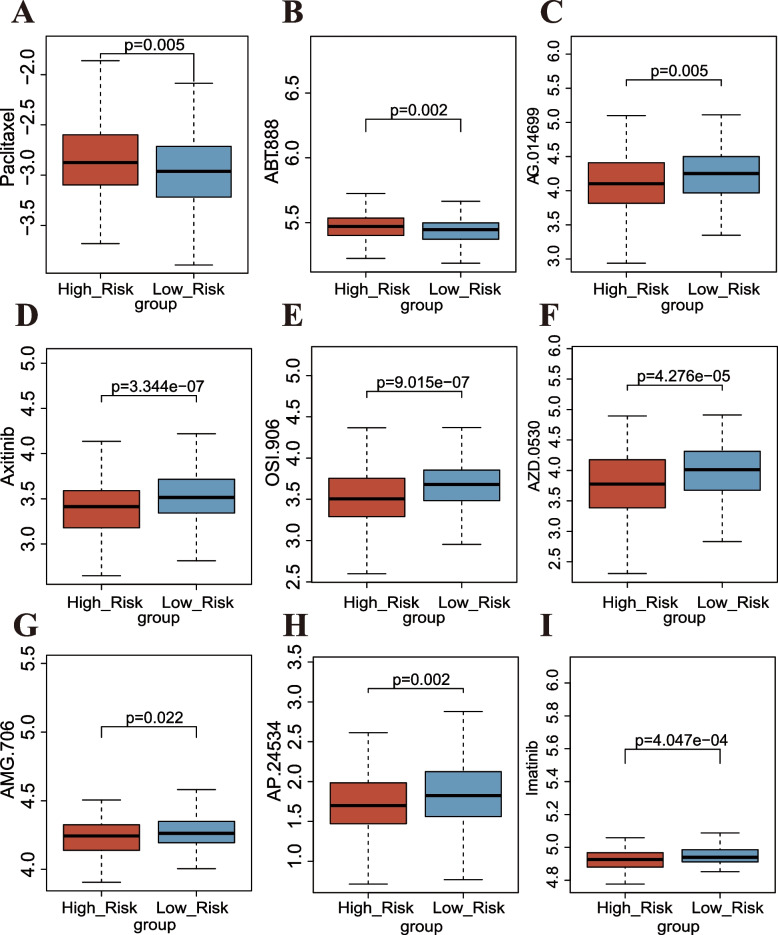
Analysis of drug sensitivity for the MRL-model. **A**-**I** Relationships between risk scores and IC50 level of Paclitaxel (**A**), ABT.888 (**B**), AG.014699 (**C**), Axitinib (**D**), OSI.906 (**E**), AZD.0530 (**F**), AMG.706 (**G**), AP.24534 (**H**), and Imatinib (**I**)

Based on data from GDSC database, the Spearman’s correlation coefficients between drug susceptibility and expression levels of the eight MRLs in the risk model was calculated (Supplementary Fig. S10A). Our data showed that AC010761.10 was highly expressed and resistant to most drugs (such as bleomycin) and the level of USP30-AS1 was negatively correlated with several drugs (such as paclitaxel). The results provided new insights into the molecular resistance mechanisms of these MRLs. We found that the IC50 levels of Paclitaxel (*P* = 0.005) and ABT.888 (Veliparib, *P* = 0.002) in the high-risk groups were observably higher than those in the low-risk group, suggesting a negative correlation between risk score and the drug susceptibility (Fig. 10A, B). Nevertheless, the exact opposite was observed regarding AG.014699 (Rucaparib, *P* = 0.005) (Fig. 10C), Axitinib (*P* = 3.344e-07) (Fig. 10D), OSI.906 (Linsitinib, *P* = 9.015e-07) (Fig. 10E), AZD.0530 (Saracatinib, *P* = 4.276e-05) (Fig. 10F), AMG.706 (Motesanib, *P* = 0.022) (Fig. 10G), AP.24534 (Ponatinib, *P* = 0.002) (Fig. 10H), and Imatinib (*P* = 4.047e-04) (Fig. 10I). Besides, other drugs commonly used in OC chemotherapy, such as Cisplatin (*P* = 0.248), Bleomycin (*P* = 0.347), Gemcitabine (*P* = 0.32), and Vinorelbine (*P* = 0.848) showed no difference between the two subgroups (Supplementary Fig. S10B-E). The results suggest that chemotherapy drugs have different clinical implications for OC patients with different risk score and OC patients need personalized treatment.

### Construction of ceRNA network

**Fig. 11:**
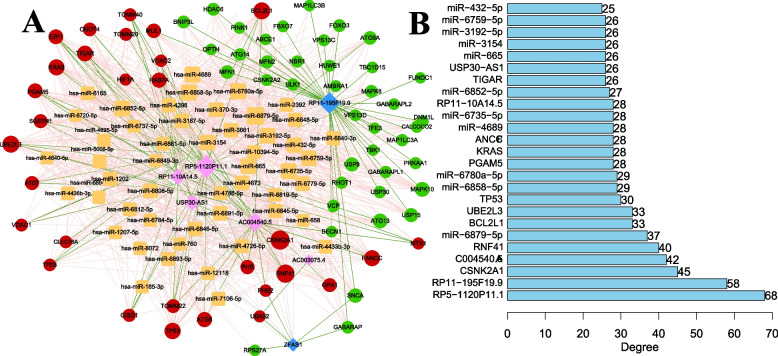
Construction of ceRNA network. **A** ceRNA network. Circle represents differential MRG, red represents up-regulation, green represents down-regulation. Diamond represents differential MRL, pink represents up-regulation, blue represents down-regulation. Green line represents positive correlation. Orange square represents predicted miRNA, gray line represents competitive binding of lncRNA to miRNA. Pink line indicates that miRNA regulate mRNA. **B** The top 25 genes with high connectivity

We predicted 35,007 miRNA-mRNA pairs and 878 lncRNA-miRNA pairs primitively. The lncRNA-miRNA-mRNA relationship pairs regulated by the same miRNA were further screened, and mRNA-lncRNA co-expression should be positively correlated (correlation coefficient > 0.2), thus obtaining 3668 lncRNA-miRNA-mRNA pairs. Definitively, there were 539 miRNAs, 73 mRNAs and 8 lncRNAs. Because of the large number of miRNAs, we further counted the number of miRNAs simultaneously regulating multiple lncRNA-mRNA relationship pairs. If a miRNA could simultaneously regulate multiple lncRNA-mRNA relationships, this miRNA may play an important role. Therefore, we focused on screening TOP50 miRNA and extracting its corresponding lncRNA-miRNA-mRNA relationship pair and carried out the construction of ceRNA network. It can be seen Fig. 11A, the network consisted of 7 lncRNAs, 50 miRNA and 71 mRNA. The network contained 122 lncRNA-mRNA co-expression pairs, 798 miRNA-mRNA pairs and 116 lncRNA-miRNA pairs. We analyzed the connectivity of each node of the network to obtain the connectivity of mRNA, miRNA and lncRNA. By ranking the connectivity of each node, RNA molecules that may play important roles were identified (Fig. 11B). The constructed ceRNA network initially described that MRLs affect mRNA expression by sharing miRNA, which provides foundations for further exploration of the regulatory mechanism of OC based on mitophagy.

## Discussion

OC has hidden early symptoms and a poor 5-year survival rate. The accuracy of OC biomarker screening is still low. OC is also a multifactorial and complex disease, and the goal of treatment is to reduce the tumor burden, prolong survival time and improve the quality of life of patients. Patients with different pathologic types receiving similar treatment may have significantly different progression free survival (PFS) and OS [61]. Traditional prognostic factors based on clinicopathological parameters are not sufficient to predict the prognosis of patients [61]. There is still controversy surrounding the existing predictive models’ ability to assess prognosis, hence, there is no marker that can accurately predict the clinical outcome of OC. Identifying OC patients with high-risk clinical outcomes and actively improving their prognosis is the focus of current research. The guidelines and consensus are gradually integrating genetic testing into the standard treatment [62]. The expression of genes associated with OC is gradually improving the situation and future prognostic models based on gene expression profiles need to be further explored. With the continuous development of technology (such as high-throughput sequencing reserved in TCGA), the methods for predicting the prognosis of OC are maturing and improving, and we can improve the standards for searching prognostic factors closely related to clinical outcomes and treatment decisions.

WGCNA analysis can help us to understand the interactions between MRGs and, ultimately, the gene networks or modules associated with mitophagy. The gene expression profiles of OC we extracted from the TCGA database provided sufficient data support for the application of WGCNA analysis in our study. Ulteriorly, we aggregated highly correlated lncRNAs into modules (correlation coefficient > 0.8). The blue module with strong positive correlation with MRGs and green module with strong negative correlation with MRGs were selected by weighted calculation of gene expression profiles several times according to the correlation coefficient and *P* value to screen the lncRNAs with high correlation with mitophagy, thereby reducing the loss of useful information. Traditional lncRNA-mRNA co-expression was calculated based on the Pearson correlation coefficient between genes and then set a hard thresholding to determine whether the network exists [63–65]. However, setting the threshold only based on Pearson can lead to the loss of real information. Different from traditional Pearson method, we used soft thresholding (R^2^ > 0.85) of WGCNA to determine whether MRLs and MRGs were associated and weighed the correlation coefficients between genes to obtain the gene co-expression matrix. The connections between genes should meet the scale-free network distribution. The expression patterns of genes in each constructed module are very similar, and hub-gene in the module helps to understand the pathogenesis of disease at the molecular level. In a word, WGCNA analysis can filter out irrelevant noisy data and find key molecular mechanisms related to mitophagy in our study. In subsequent studies, experimental methods are needed to confirm the molecular biological correlation between the MRLs we identified and mitophagy, such as mitochondrial membrane potential measurement [66], mitochondrial morphology observing, mitophagy markers detecting and so on.

Data mining based on bioinformatics can be used to explore important biological phenotypes associated with high-dimensional datasets. TCGA and GEO are databases with large-scale genomic analysis capabilities to assess molecular biological signatures associated with OC. Recent developments in next-generation sequencing technologies have greatly expanded our understanding of lncRNAs, which are more abundant in both quantity and function than mRNAs. There have been some successful cases of molecular marker screening by bioinformatics. Bioinformatics analysis based on a large sample (such as samples from TCGA or GEO) can avoid accidental factors more and has stronger generalization. However, a single bioinformatics algorithm is often used in previous studies, which may lead to excessive data perturbation and poor reliability of results [67–69]. Therefore, using dividing gene modules based on clustering, the target module needs to be selected for regression analysis to analyze the correlation degree between genes and features, thus improving the accuracy of screening lncRNA for prognosis of OC. We carried out comprehensive analyses based on bioinformatics in this study. LASSO Cox Regression analysis was carried out after WGCNA, which can improve the precision of screening prognostic MRLs and provide a basis for improving the prognosis of OC.

Non-coding RNA regulates various physiological and pathological processes in the human body. It has been confirmed that the reproductive disorders are related to non-coding RNA to some extent.

The fertility-sparing measures, including hormonal treatment, hysteroscopic resection [70], gametes cryopreservation [71], should be appropriately reserved in the treatment of early-stage or low-risk endometrial cancer (EC) [70, 72, 73], cervical cancer (CC) [74] and OC [75], and the non-coding RNA-based diagnostics and therapeutics are the valuable options for implications for the fertility-sparing process [76]. Some lncRNAs have also been reported to be involved in the anti-EC effects of progesterone, which may provide new insights into fertility-sparing process [77]. As for MRLs involved in mitophagy, knockdown lncRNA MALAT1 can reduce mitophagy in hepatocellular carcinoma [78]. The lack of methionine down-regulates LINC00079, thus activating mitophagy to inhibit cell proliferation in gastric cancer [79]. Overexpression of the peptide encoded by LINC-PINT decreases the mitophagy of hepatocellular carcinoma in vitro and in vivo [80]. In general, studies on lncRNA regulation of mitophagy are still very rare in human cancers and even blank in OC.

A single gene is often unable to predict the prognosis and treatment outcome of tumor patients accurately and stably, but the comprehensive score that integrates the contribution of multiple genes model can overcome this shortcoming. Eight optimal MRL combinations with prognostic value (log-rank test *P* < 0.05) were screened by integrating WGCNA and LASSO analyses in our study, including AC003075.4 (HR < 1), AC004540.5 (HR < 1), AC010761.10 (HR < 1), RP5-1120P11.1 (HR < 1), RP11-10A14.5 (HR < 1), USP30-AS1 (HR < 1), RP11-195F19.9 (HR > 1), and ZFAS1 (HR > 1). We also found the difference of the eight MRLs between normal and cancer tissues via Quantitative Real-time PCR experiments and TCGA. Hence, the differentially expressed and prognostic MRLs can be used as diagnostic markers to identify cancer and non-cancer as well as be expected to be involved in OC progression and deserve further study. RP5-1120P11.1 was identified to participate in proliferation, cycle regulation, and invasion of OC cells [81]. ZFAS1 has been reported to be involved in biological functions of OC. To be specific, ZFAS1 could regulate OC cell malignancy through ZFAS1/miR-150-5p/Sp1 axis [82]; ZFAS1 could also regulates metastasis and platinum resistance [83] of OC via let-7a/BCL-XL/S axis [84]. For other tumors, it was reported that the repression of mitophagy mediated by lncRNA USP30-AS1 could lead to glioblastoma tumorigenesis [85]. USP30-AS1 was proven to regulate the mass and protein expression of mitochondria, thus mediating mitophagy in glioblastoma cells [85]. Mengyue Chen et al. determined the molecular mechanisms of USP30-AS1/miR-299-3p/PTP4A1 axis in CC malignancy [86]. In acute myeloid leukemia, USP30-AS1 may be a regulator of cancer cell survival [87]. ZFAS1 has been widely proved to be related with the development and progression of human cancers [88], including colorectal cancer [89, 90], nasopharyngeal carcinoma [91], oral squamous cell carcinoma [92], pancreatic cancer [93], and so on. Therefore, the study of the biological relevance of MRLs to OC is still in its infancy, and biological experiments are needed to prove how these MRLs play the role of mitophagy in OC. Besides, our constructed ceRNA network initially described the regulatory mechanism of mitophagy, which needs experimental validation, such as Dual-Luciferase Reporter Assay.

Recognition of prognostic factors and characterization of the molecular classification has great significance in physiology, pathology, treatments, and clinical trials for gynecologic malignant tumors [94, 95]. Accurate prognosis assessment and stratified management of OC patients is the key to improve patient survival. Using multiple influencing factors to establish a prognostic model for OC has been attempted in the past decade with unsatisfactory results. Previous research have established prognostic indexes for patients with OC, including FIGO stage, residual lesion size, histological grade, and ascites [96]. However, the study failed to be generalized due to its small sample size and short follow-up time. Although some clinicopathological parameters affect the prognosis of OC patients have reached some consensus in clinical treatment [97], no recognized diagnostic guidelines have clearly pointed out. Therefore, there is still a long way to go to construct and standardize the prognostic model of OC and popularize it in clinic. Herein, we established the prognostic model based on the eight optimal MRL combinations. The prognosis and independent prognostic value of the MRL-model was verified and validated in TCGA and GEO databases using K-M (log-rank test *P* < 0.0001; log-rank test *P* = 0.012), Univariate Cox regression (*P* < 0.001, HR = 1.960, 95% CI:1.520–2.528; *P* = 0.011, HR = 1.439, 95% CI:1.086–1.906), and Multivariate Cox regression analyses (*P* < 0.001, HR = 1.795, 95% CI:1.371–2.350; *P* = 0.038, HR = 1.349, 95% CI:1.017–1.789), indicating the generalisability. Although there have been articles published on prognostic models based on mRNAs [67–69, 98] or the prognostic models established using clinicopathological factors [99, 100] for OC, none of these prognostic models has been externally validated. The prognostic models were controversy surrounding the existing predictive ability to assess prognosis and there are no uniform prognostic models in clinical practice. It is worth mentioning that the MRL-model can also stratify OC patients with different prognostic risks in consideration of clinicopathological parameters, including ethnic and demographic factors (White or Nonwhite). Our study proposes a new prognostic MRL-model whose clinical applicability deserves further exploration. We also detected the expression of several MRLs in the prognostic MRL-model in tissues, which can also lay a foundation for subsequent studies on lncRNA regulation of mitophagy to a certain extent.

The Nomogram can integrate various clinicopathological parameters to evaluate the possibility of occurrence of clinical events, assign and sum different influencing factors, and show them graphically [101]. In our study, we found that the prognostic MRL-model we established was superior to other clinicopathological parameters in predicting OC survival. Further, the actual OS probability and the predicted OS probability of the Nomogram was validated by discrimination and calibration, which indicated that the Nomogram can be used to quantify risk and assess prognosis in patients with OC by combining multiple factors to determine prognosis.

In addition, we compared the discrepancies between the two risk groups based on the MRL-model in functional pathways (*P* < 0.01), TMB, somatic mutation features, immunity features (Wilcox test, *P* < 0.05), chemotherapeutic drug sensitivity (Wilcox test, *P* < 0.05). Our results suggested that OC patients in the two risk groups differed at the level of immune cells. The tumor immune microenvironment is heterogeneous between patients and tumor types, and these differences in composition may suggest different barriers to anti-tumor immunity that affect a patient’s response to specific immunotherapies [102]. It is necessary to look for heterogeneity of OC patients, stratify the population benefit most from immunotherapy. We also found that the TMB level was higher in low-risk group than in high-risk group (Wilcox test, *P* < 0.05), implying that patients with lower risk score may benefit from immunotherapy. OC patients with low risk score may be more sensitive to immune checkpoint blockade therapy was further confirmed by TIDE score (Wilcox test, *P* < 0.05) and subclass mapping (Bonferroni corrected *P* = 0.01). Cytoreduction surgeries along with neoadjuvant chemotherapy are modern therapeutic regimens for advanced-stage OC, nevertheless, its safety and efficacy still need to be explored [103]. Poly (ADP-ribose) polymerase inhibitors (PARP inhibitors) showed particular benefit for OC patients [104]. Since several patients develop resistance to chemotherapy and PARP inhibitors, we need to further identify effective patient subgroups. Drug susceptibility analysis was implemented, and the results showed that OC patients in the high-risk group were resistant to Paclitaxel (*P* = 0.005) and Veliparib (*P* = 0.002), while patients with low risk score were resistant to Rucaparib (*P* = 0.005), Axitinib (*P* = 3.344e-07), Linsitinib (*P* = 9.015e-07), Saracatinib (*P* = 4.27e-5), Motesanib (*P* = 0.022), Ponatinib (*P* = 0.002), and Imatinib (*P* = 4.047e-4). For patients who are not sensitive to anti-cancer drugs, the treatment regimen should be changed in time to improve the prognosis of patients to a greater extent. However, there is a lack of indicators that can indicate drug reactivity for clinical quality decision making. Therefore, the prognostic MRL-model we established has a certain suggestive effect on the drug sensitivity of OC patients, but further validation using clinical samples is needed. The mechanism of direct or indirect influence of MRL on drug sensitivity is also worthy of further study using experimental validation.

Our study hopes to provide feasible ideas for prognosis screening and precise treatment of OC patients by constructing prognostic risk model. However, there are still some limitations of this study worth mentioning. This study is a retrospective study, which has inherent bias inevitably, such as selection bias that may occur when incomplete information is excluded. The factors that affect the prognosis of OC are complex and diverse, and more clinical factors are not included in the study due to lack of data (such as ethnic and demographic factors). Hence, we are considering including more than 300 Chinese patients to validate the MRL-model. We established the prognostic MRL-model based on the data sources from public databases, and the prognosis and independence of the MRL-model was identified and validated in TCGA and the external GEO datasets. However, the sample size still needs to be expanded and analysis based on mRNA expression profile (such as drug sensitivity) could not be carried out on GEO dataset due to lack of data. Hence, more clinical tissues are needed to verify the reliability of prediction after follow-up. Although the expression levels of eight optimal MRLs in the prognostic MRL-model were examined in the clinical samples we collected, insufficient sample size and lack of clinical data need to be further addressed to make the evidence more solid. The specific mechanism of lncRNA related to mitophagy identified by us has not been developed yet by wet experiment, which needs to be addressed in future studies.

## Conclusion

The comprehensive analysis of MRGs and MRLs revealed their roles in expression, prognosis, chemotherapy, immunotherapy and molecular mechanism of OC. By analyzing prognosis, functional pathways, mutation, immunity features, immunotherapy, and drug susceptibility, our findings demonstrated the molecular and clinical significance of the MRL-model, thus stratifying patients with high risk and improving clinical outcomes for OC patients. The MRL-based model we constructed and validated deserves further study for future clinical application after addressing the limitations, such as insufficient sample size, missing demographic factors, lack of external validation and wet experiments.

### Supplementary Information

**Additional file 1.**

**Additional file 2.**

## Data Availability

The RNA sequencing profiles and clinical information of ovary and ovarian cancer can be gained from UCSC-Xena (https://xenabrowser.net/datapages/?dataset=TcgaTargetGtex_rsem_isoform_tpm&host=https%3A%2F%2Ftoil.xenahubs.net&removeHub=http%3A%2F%2F127.0.0.1%3A7222) platform. The RNA sequencing profiles and clinical information of ovarian cancer from Gene Expression Omnibus (GEO) database are available at the following link: GSE19829 (https://www.ncbi.nlm.nih.gov/geo/query/acc.cgi?acc=GSE19829), GSE26193 (https://www.ncbi.nlm.nih.gov/geo/query/acc.cgi?acc=GSE26193), GSE30161 (https://www.ncbi.nlm.nih.gov/geo/query/acc.cgi?acc=GSE30161), and GSE63885 (https://www.ncbi.nlm.nih.gov/geo/query/acc.cgi?acc=GSE63885). The gene annotation information is available at GENCODE database (https://www.gencodegenes.org/). The mitophagy-related genes can be gained from GeneCards (https://www.genecards.org). The R packages can be acquired or installed from CRAN (https://cran.r-project.org/mirrors.html), Biocductor (https://www.bioconductor.org/), GitHub (https://github.com/GitHub), or native software R Studio (https://posit.co/downloads/). Further inquiries can be directed to the corresponding author.

## Abbreviations

OC: Ovarian cancer
lncRNA: Long non-coding RNA
CA125: Carbohydrate Antigen 125
HE4: Human Epididymis Protein 4
ROMA: Risk of Ovarian Malignancy Algorithm
TCGA: The Cancer Genome Atlas
GEO: Gene Expression Omnibus
MRL: Mitophagy-relate lncRNA
MRG: Mitophagy-relate gene
WGCNA: Weighted coexpression network analysis
LASSO: Least absolute shrinkage and selection operator
OS: Overall survival
TMB: Tumor mutational burden
MAF: Mutation Annotation Format
FDR: False discovery rate
K-M: Kaplan–Meier
PPI: Protein-protein interaction
GSEA: Gene set enrichment analysis
ssGSEA: Single-sample gene set enrichment analysis
GSVA: Gene set variation analysis
CIBERSORT: Cell-type Identification By Estimating Relative Subsets Of RNA Transcripts
MCPcounter: Microenvironment Cell Populations-counter
ESTIMATE: Estimation of STromal and Immune cells in MAlignant Tumor tissues using Expression data
ICB: Immune checkpoint blockade
TIDE: Tumor immune dysfunction and exclusion
GDSC: Genomics of Drug Sensitivity in Cancer
ceRNA: competing endogenous RNA
HR: Hazard ratio
CI: Confidence interval
GO: Gene Ontology
PFS: Progression free survival
EC: Endometrial cancer
CC: Cervical cancer
