# Supplementary material for: Comprehensive analyses of mitophagy-related genes and mitophagy-related lncRNAs for patients with ovarian cancer
Source: BMC Womens Health. 2024 Jan 13;24:37. doi: 10.1186/s12905-023-02864-5 (PMC10788026; doi:10.1186/s12905-023-02864-5)
Supplement: Supplementary file 1 — Additional file 1. [file 12905_2023_2864_MOESM1_ESM.pdf]

Supplemental information

Comprehensive analyses of mitophagy-related genes and mitophagy-related lncRNAs for patients with ovarian cancer

Jianfeng Zheng[1†], Shan Jiang[1†], Xuefen Lin[1†], Huihui Wang[2], Li Liu[1], Xintong Cai[1], Yang Sun[1, *]

[1]Department of Gynecology, Clinical Oncology School of Fujian Medical University, Fujian Cancer Hospital, Fuzhou, 350014, China

[2]Department of Anesthesiology, The Central hospital of Wenzhou City, 32 Dajian Lane, Wenzhou 325000, China

*** Correspondence:**

Correspondence should be addressed to Yang Sun.

Address: No.420, Fuma Road, Jin ‘an District, Fuzhou City, Fujian Province, P. R. China

Email: sunyang@fjzlhospital.com

[†]Jianfeng Zheng, Shan Jiang and Xuefen Lin contributed equally to this work.

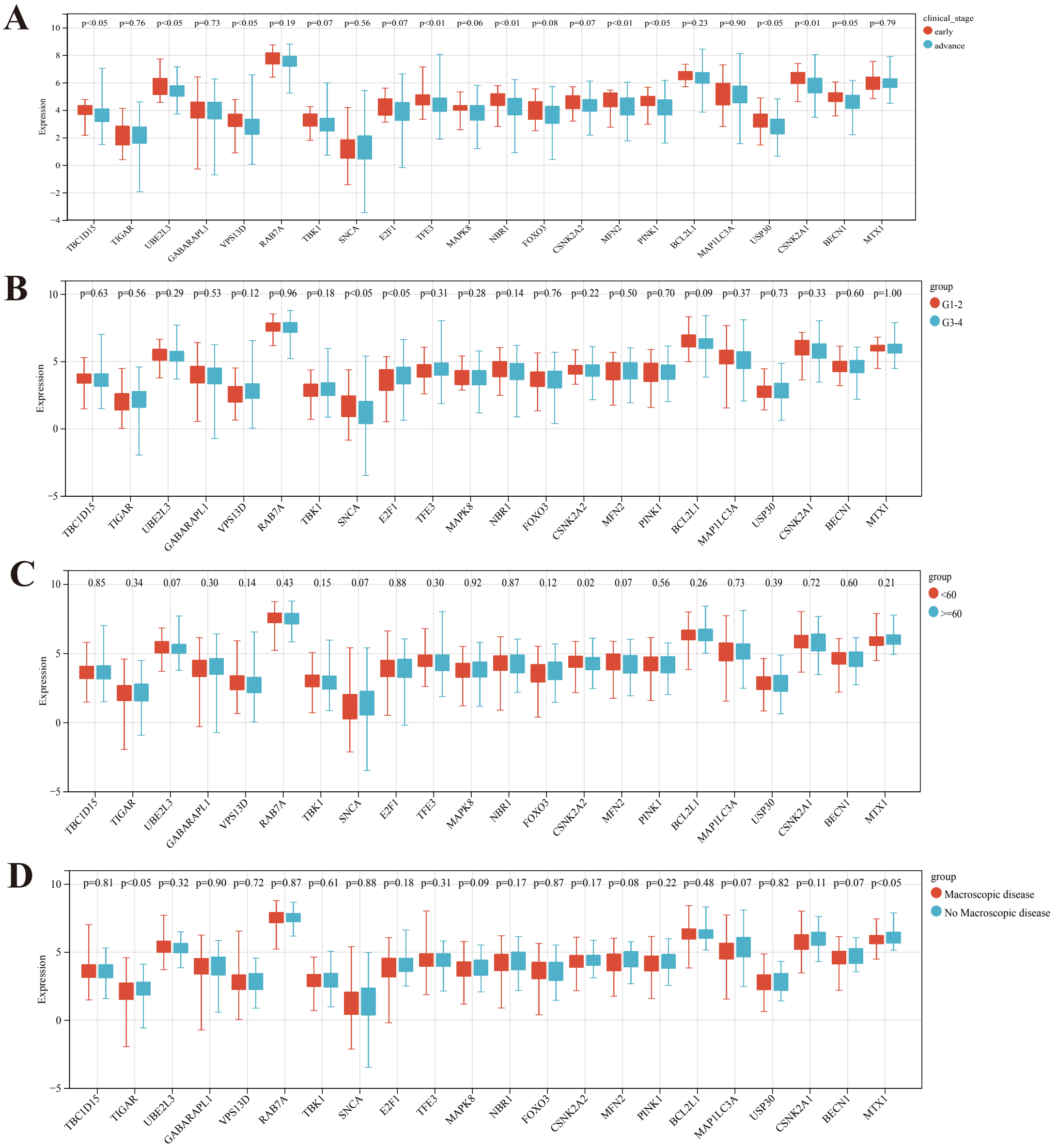

Supplementary Figure S1. The relationship between the 22 prognostic MRGs and clinical characteristics for Stage **(A)**, Grade **(B)**, Age **(C)**, and Macroscopic disease **(D)**.

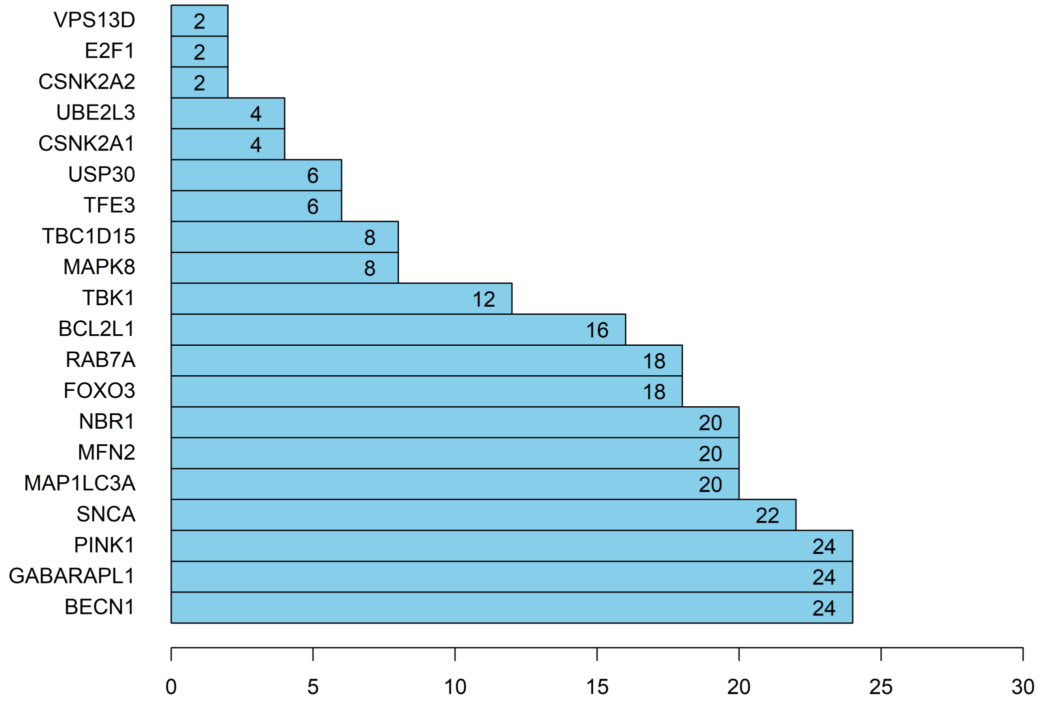

Supplementary Figure S2. The top 20 hub genes among the prognostic MRGs. The number indicates the number of nodes.

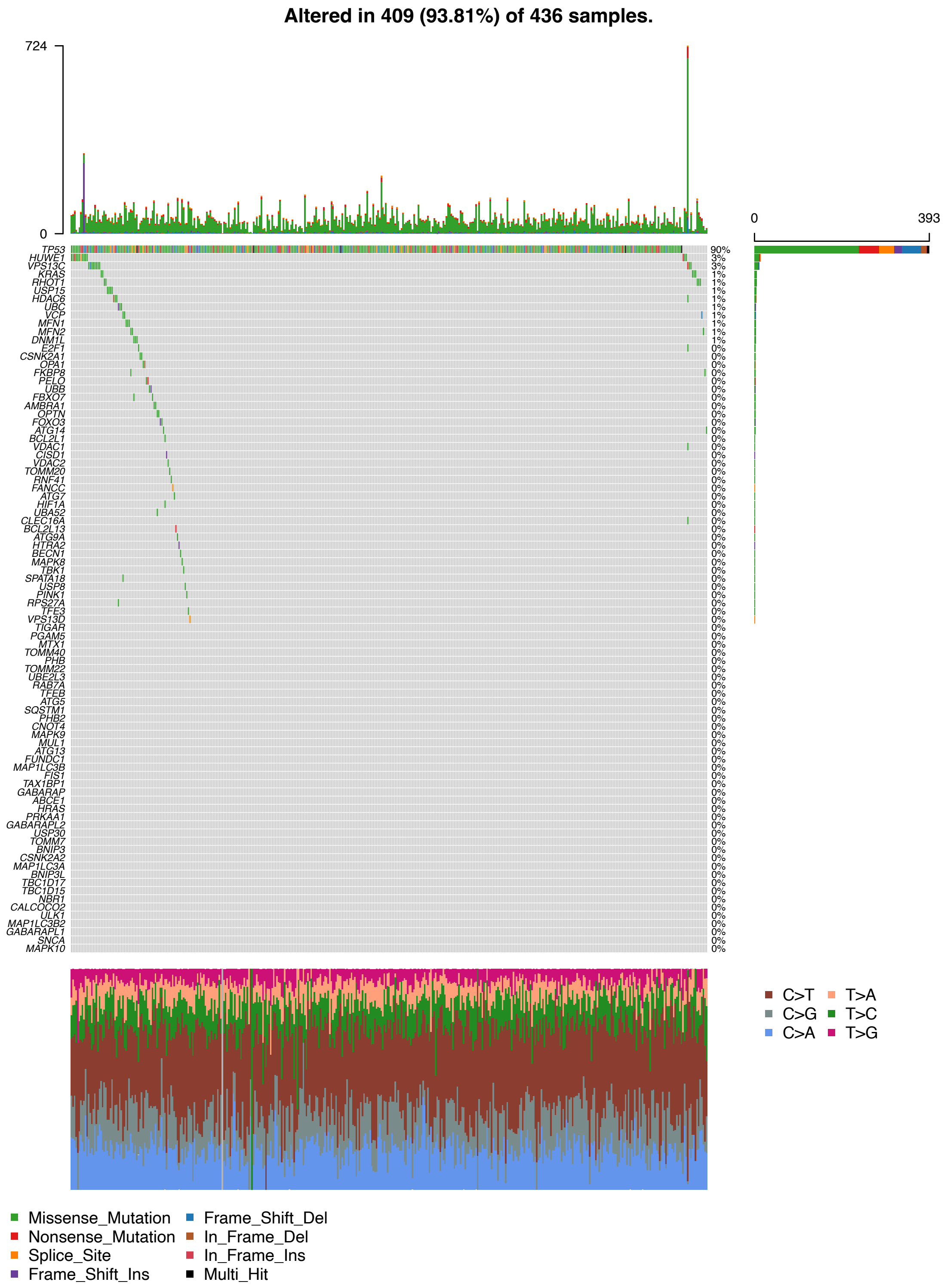

Supplementary Figure S3. Mutation frequency of MRGs in TCGA-OV cohort. Each column represented an individual patient. The upper barplot showed TMB, the number on the right

indicated the mutation frequency in each gene. The right barplot showed the proportion of each variant type.

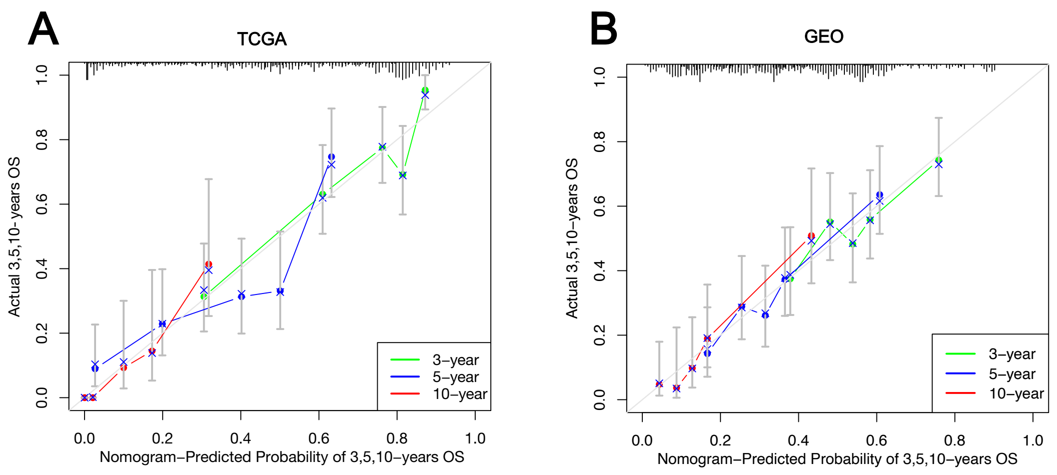

Supplementary Figure S4. The discrimination and calibration of the Nomogram showing the actual OS probability and the predicted OS probability for TCGA training **(A)** and GEO validation **(B)** datasets. The closer the predicted curve is to 45°, the better the prediction ability.

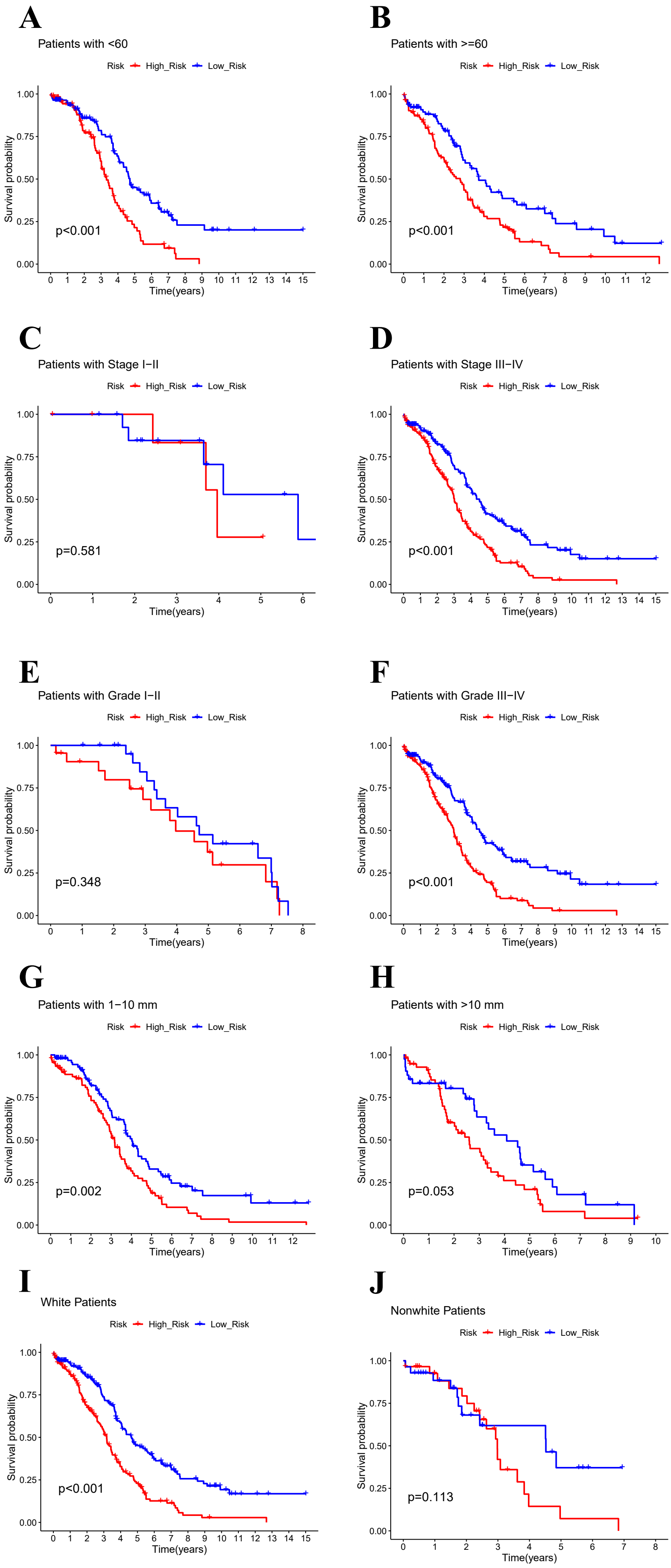
A
Patients with <60
Risk High_Risk Low_Risk
Survival probability
Time(years)
p<0.001
B
Patients with >=60
Risk High_Risk Low_Risk
Survival probability
Time(years)
p<0.001
C
Patients with Stage I−II
Risk High_Risk Low_Risk
Survival probability
Time(years)
p=0.581
D
Patients with Stage III−IV
Risk High_Risk Low_Risk
Survival probability
Time(years)
p<0.001
E
Patients with Grade I−II
Risk High_Risk Low_Risk
Survival probability
Time(years)
p=0.348
F
Patients with Grade III−IV
Risk High_Risk Low_Risk
Survival probability
Time(years)
p<0.001
G
Patients with 1−10 mm
Risk High_Risk Low_Risk
Survival probability
Time(years)
p=0.002
H
Patients with >10 mm
Risk High_Risk Low_Risk
Survival probability
Time(years)
p=0.053
I
White Patients
Risk High_Risk Low_Risk
Survival probability
Time(years)
p<0.001
J
Nonwhite Patients
Risk High_Risk Low_Risk
Survival probability
Time(years)
p=0.113

Supplementary Figure S5. The survival curves of the MRL-model stratified by clinicopathological parameters. **(A)** Age<60 years. **(B)** Age>=60 years. **(C)** Stage I-II. **(D)** Stage III-IV. **(E)** Grade I-II. **(F)** Grade III-IV. **(G)** Tumor Residual Disease 1-10mm. **(H)** Tumor Residual Disease >10mm. **(I)** White. **(J)** Nonwhite. The *P* values are tested by log-rank.

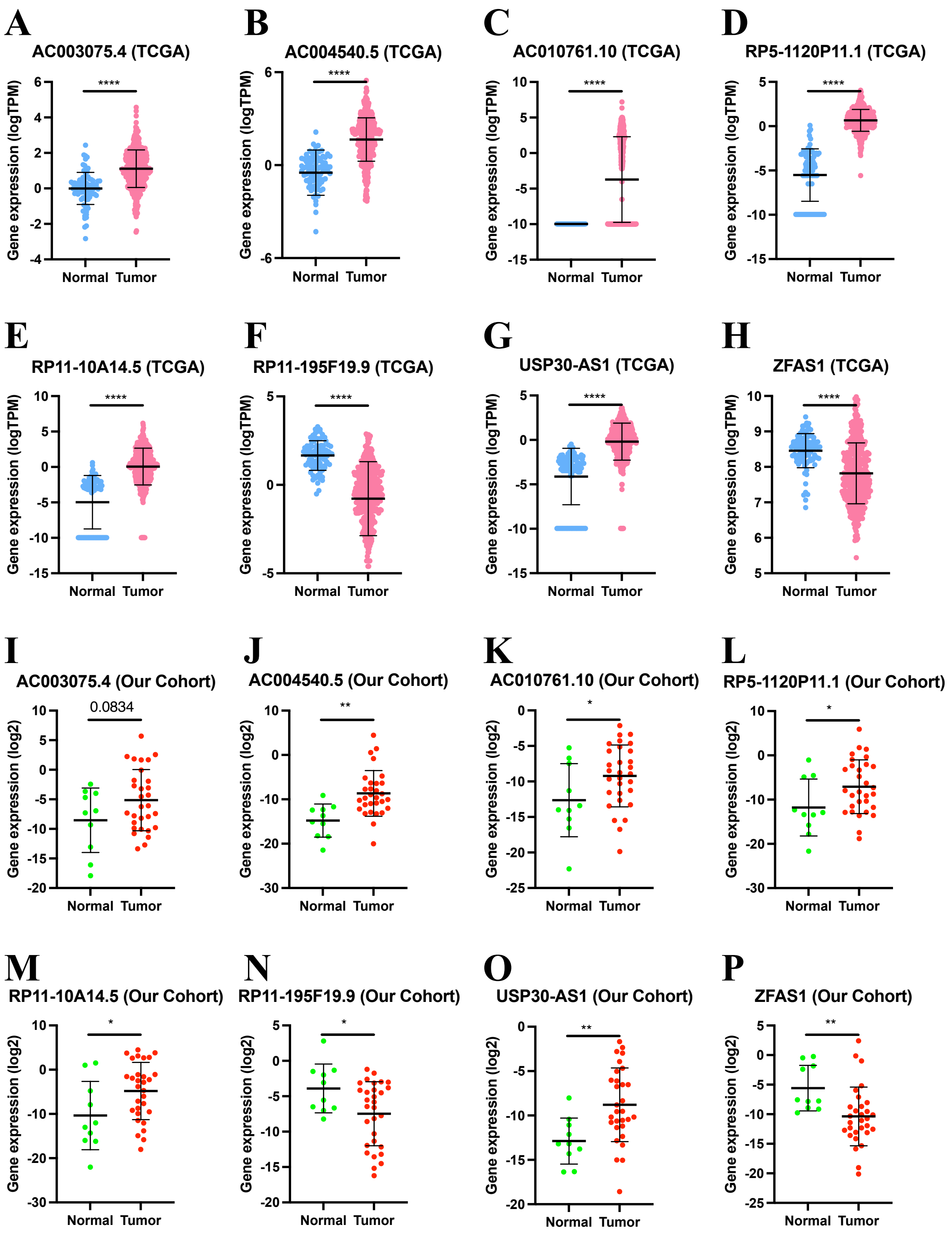

Supplementary Figure S6. Expression values of MRLs included in the MRL-based risk model.

(A-H) Expression values of MRLs in TCGA for AC003075.4 **(A)**, AC004540.5 **(B)**, AC010761.10 **(C)**, RP5-1120P11.1 **(D)**, RP11-10A14.5 **(E)**, RP11-195F19.9 **(F)**, USP30-AS1 **(G)** and ZFAS1 **(H)**. **(I-P)** Expression values of MRLs in our cohort for AC003075.4 **(I)**, AC004540.5 **(J)**, AC010761.10 **(K)**, RP5-1120P11.1 **(L)**, RP11-10A14.5 **(M)**, RP11-195F19.9 **(N)**, USP30-AS1 **(O)** and ZFAS1 **(P)**. *P* values: ns, not significant; **P*< 0.05; ***P*< 0.01; ****P*< 0.001, *****P*< 0.0001.

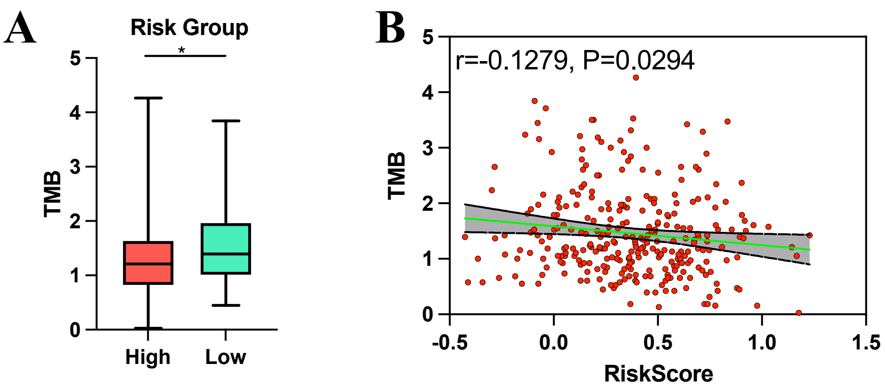

Supplementary Figure S7. Evaluation of TMB level between the two risk groups. **(A)** TMB differences between risk groups. **(B)** The correlation between risk score and TMB level. **P*< 0.05.

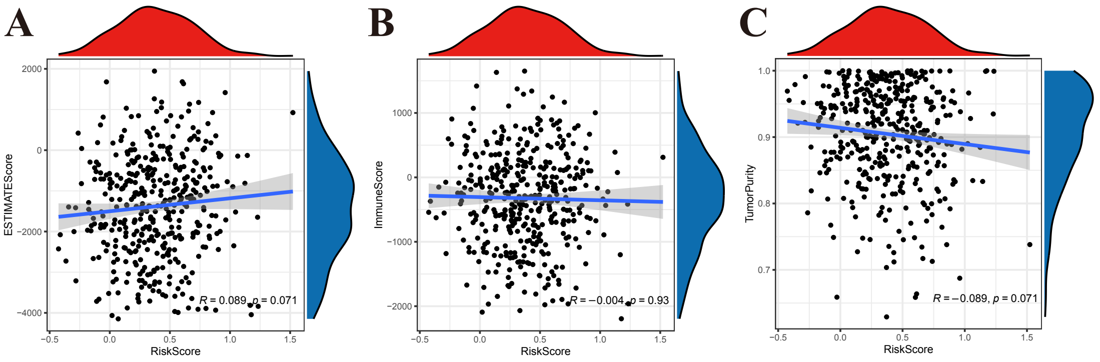

Supplementary Figure S8. The correlation between risk score and ESTIMATE Score **(A)**, Immune Score **(B)**, Tumor Purity **(C)**.

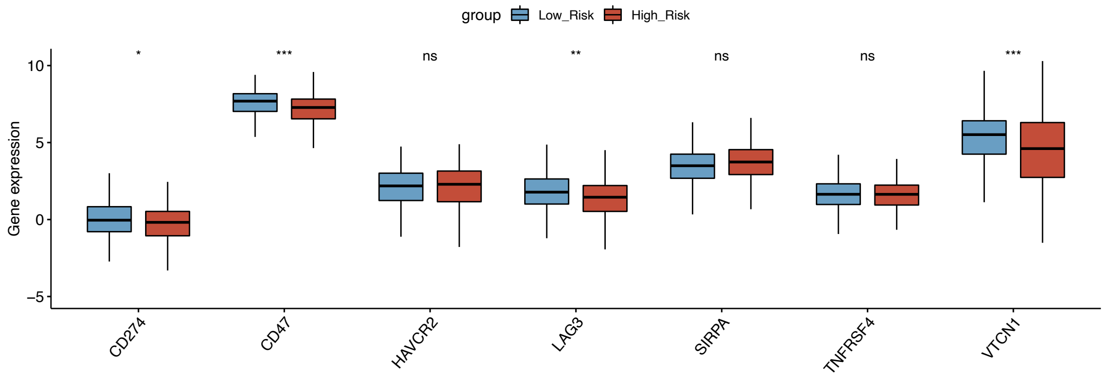

Supplementary Figure S9. Expression of immune checkpoints in the low and high-risk groups. P values: ns, not significant; *P< 0.05; **P< 0.01; ***P< 0.001, ****P< 0.0001.

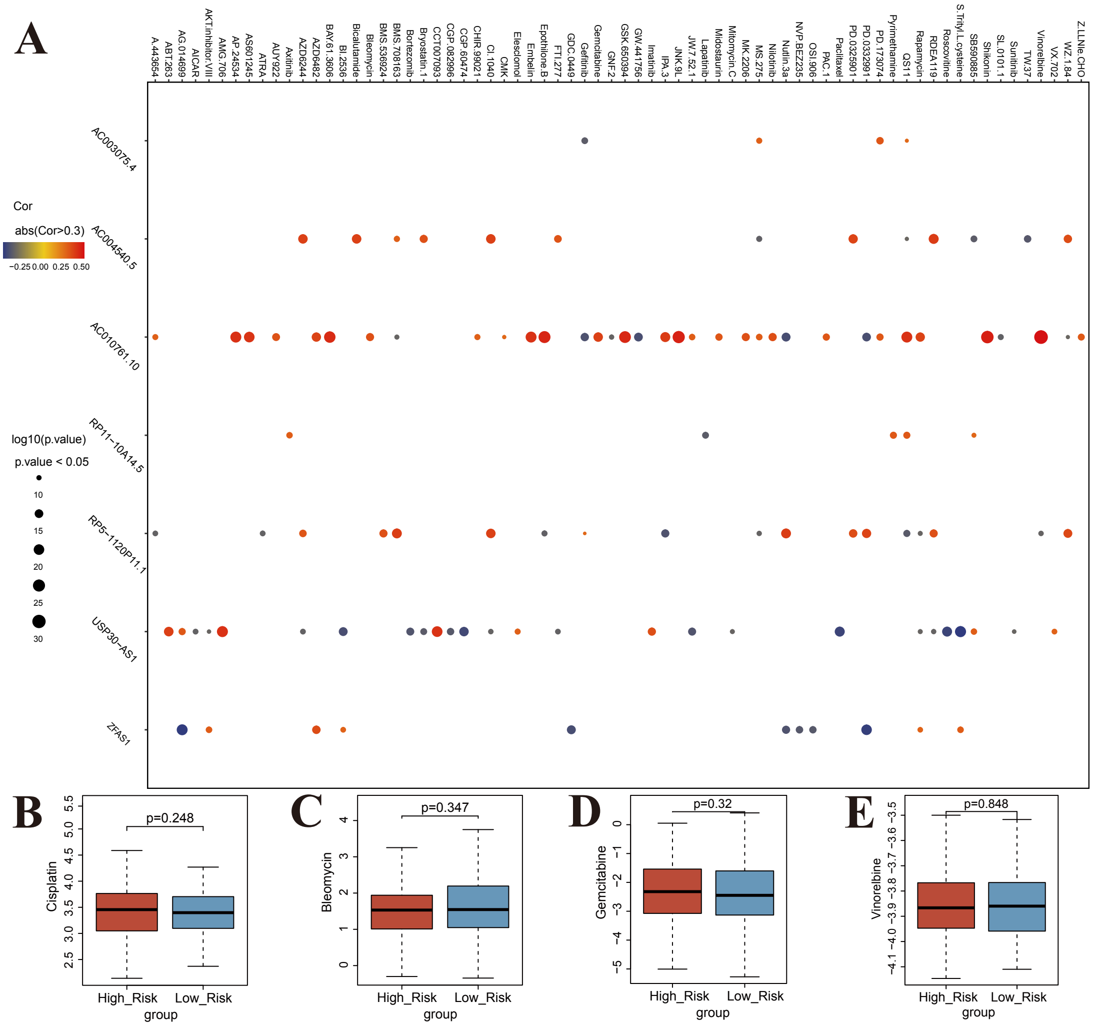

Supplementary Figure S10. Analysis of chemotherapeutic sensitivity based on the MRL-based risk model. Correlation between MRLs and the IC50 of drug in OC. The bubble color indicated the

degree of correlation index. The bubble size indicated the *P* value. **(B-E)** Relationships between risk scores and IC50 level of Cisplatin **(B)**, Bleomycin **(C)**, Gemcitabine **(D)**, and Vinorelbine **(E)**.
