# Supplementary material for: Comprehensive analyses of mitophagy-related genes and mitophagy-related lncRNAs for patients with ovarian cancer
Source: BMC Womens Health. 2024 Jan 13;24:37. doi: 10.1186/s12905-023-02864-5 (PMC10788026; doi:10.1186/s12905-023-02864-5)
Supplement: Supplementary file 2 — Additional file 2. [file 12905_2023_2864_MOESM2_ESM.pdf]

Supplemental information

Comprehensive analyses of mitophagy-related genes and mitophagy-related lncRNAs for patients with ovarian cancer

Jianfeng Zheng[1†], Shan Jiang[1†], Xuefen Lin[1†], Huihui Wang[2], Li Liu[1], Xintong Cai[1], Yang Sun[1, *]

[1]Department of Gynecology, Clinical Oncology School of Fujian Medical University, Fujian Cancer Hospital, Fuzhou, 350014, China

[2]Department of Anesthesiology, The Central hospital of Wenzhou City, 32 Dajian Lane, Wenzhou 325000, China

*** Correspondence:**

Correspondence should be addressed to Yang Sun.

Address: No.420, Fuma Road, Jin ‘an District, Fuzhou City, Fujian Province, P. R. China

Email: sunyang@fjzlhospital.com

[†]Jianfeng Zheng, Shan Jiang and Xuefen Lin contributed equally to this work.

Table S 1. The list of software, packages and versions

**Software or Packages**	**Version**
R	3.6.1
limma	3.10.3
survminer	0.4.3
Cytoscape	3.4.0
maftools	2.16.0
WGCNA	1.61
glmnet	2.0-18
sva	3.48.0
rms	6.7.0
GSEA	4.3.2
GSVA	1.48.3
CIBERSORT	0.1.0
xCELL	1.1.0
MCPcounter	1.2.0
ESTIMATE	1.1.7
pRRophetic	0.5
miranda	3.3a
CytoNCA	2.1.6
